# Risk of depression, suicide and psychosis with hydroxychloroquine treatment for rheumatoid arthritis: a multinational network cohort study

**DOI:** 10.1093/rheumatology/keaa771

**Published:** 2020-12-25

**Authors:** Jennifer C E Lane, James Weaver, Kristin Kostka, Talita Duarte-Salles, Maria Tereza F Abrahao, Heba Alghoul, Osaid Alser, Thamir M Alshammari, Carlos Areia, Patricia Biedermann, Juan M Banda, Edward Burn, Paula Casajust, Kristina Fister, Jill Hardin, Laura Hester, George Hripcsak, Benjamin Skov Kaas-Hansen, Sajan Khosla, Spyros Kolovos, Kristine E Lynch, Rupa Makadia, Paras P Mehta, Daniel R Morales, Henry Morgan-Stewart, Mees Mosseveld, Danielle Newby, Fredrik Nyberg, Anna Ostropolets, Rae Woong Park, Albert Prats-Uribe, Gowtham A Rao, Christian Reich, Peter Rijnbeek, Anthony G Sena, Azza Shoaibi, Matthew Spotnitz, Subbian Vignesh, Marc A Suchard, David Vizcaya, Haini Wen, Marcel de Wilde, Junqing Xie, Seng Chan You, Lin Zhang, Simon Lovestone, Patrick Ryan, Daniel Prieto-Alhambra, for the OHDSI-COVID-19 consortium

**Affiliations:** 1 Centre for Statistics in Medicine, Nuffield Department of Orthopaedics, Rheumatology, and Musculoskeletal Sciences (NDORMS), University of Oxford, Oxford, UK; 2 Janssen Research and Development, Titusville, NJ, USA; 3 Real World Solutions, IQVIA, Cambridge, MA, USA; 4 Fundació Institut Universitari per a la recerca a l’Atenció Primària de Salut Jordi Gol i Gurina (IDIAPJGol), Barcelona, Spain; 5 Faculty of Medicine, University of Sao Paulo, Sao Paulo, Brazil; 6 Faculty of Medicine, Islamic University of Gaza, Gaza, Palestine; 7 Massachusetts General Hospital, Harvard Medical School, Boston, MA, USA; 8 Medication Safety Research Chair, King Saud University, Riyadh, Saudi Arabia; 9 Nuffield Department of Clinical Neurosciences, University of Oxford, Oxford, UK; 10 Actelion Pharmaceuticals, Allschwil, Switzerland; 11 Georgia State University, Atlanta, GA, USA; 12 Real-World Evidence, Trial Form Support, Barcelona,Spain; 13 School of Medicine, Andrija Štampar School of Public Health, University of Zagreb, Zagreb, Croatia; 14 Department of Biomedical Informatics, Columbia University Irving Medical Center, New York, NY, USA; 15 New York-Presbyterian Hospital, New York, NY, USA; 16 Clinical Pharmacology Unit, Zealand University Hospital, Roskilde, Denmark; 17 NNF Centre for Protein Research, University of Copenhagen, Copenhagen, Denmark; 18 Real World Science & Digital, AstraZeneca, Cambridge, UK; 19 Department of Veterans Affairs, Salt Lake City, UT, USA; 20 University of Utah School of Medicine, Salt Lake City, UT, USA; 21 College of Medicine, University of Arizona, Tucson, AZ, USA; 22 Division of Population Health and Genomics, University of Dundee, Dundee, UK; 23 Department of Medical Informatics, Erasmus University Medical Center, Rotterdam, The Netherlands; 24 Department of Psychiatry, University of Oxford, Oxford, UK; 25 School of Public Health and Community Medicine, Institute of Medicine, Sahlgrenska Academy, University of Gothenburg, Gothenburg, Sweden; 26 Department of Biomedical Informatics, Ajou University School of Medicine, Suwon-si, Gyeonggi-do, South Korea; 27 College of Engineering, University of Arizona, Tucson, AZ, USA; 28 Departments of Biomathematics and Human Genetics David Geffen School of Medicine at UCLA, and Department of Biostatistics, UCLA School of Public Health, South Los Angeles, CA, USA; 29 Bayer Pharmaceuticals, Sant Joan Despi, Barcelona, Spain; 30 Department of Pharmacy, Shanghai Chest Hospital, Shanghai Jiao Tong University, Shanghai, P.R. China; 31 School of Public Health, Peking Union Medical College, Chinese Academy of Medical Sciences, Beijing, P.R. China; 32 Melbourne School of Population and Global Health, University of Melbourne, Melbourne, Australia; 33 Janssen-Cilag, 50-100 Holmers Farm Way, High Wycombe HP12 4EG, UK

**Keywords:** HCQ, safety, epidemiology, RA, psychosis, depression

## Abstract

**Objectives:**

Concern has been raised in the rheumatology community regarding recent regulatory warnings that HCQ used in the coronavirus disease 2019 pandemic could cause acute psychiatric events. We aimed to study whether there is risk of incident depression, suicidal ideation or psychosis associated with HCQ as used for RA.

**Methods:**

We performed a new-user cohort study using claims and electronic medical records from 10 sources and 3 countries (Germany, UK and USA). RA patients ≥18 years of age and initiating HCQ were compared with those initiating SSZ (active comparator) and followed up in the short (30 days) and long term (on treatment). Study outcomes included depression, suicide/suicidal ideation and hospitalization for psychosis. Propensity score stratification and calibration using negative control outcomes were used to address confounding. Cox models were fitted to estimate database-specific calibrated hazard ratios (HRs), with estimates pooled where I^2^ <40%.

**Results:**

A total of 918 144 and 290 383 users of HCQ and SSZ, respectively, were included. No consistent risk of psychiatric events was observed with short-term HCQ (compared with SSZ) use, with meta-analytic HRs of 0.96 (95% CI 0.79, 1.16) for depression, 0.94 (95% CI 0.49, 1.77) for suicide/suicidal ideation and 1.03 (95% CI 0.66, 1.60) for psychosis. No consistent long-term risk was seen, with meta-analytic HRs of 0.94 (95% CI 0.71, 1.26) for depression, 0.77 (95% CI 0.56, 1.07) for suicide/suicidal ideation and 0.99 (95% CI 0.72, 1.35) for psychosis.

**Conclusion:**

HCQ as used to treat RA does not appear to increase the risk of depression, suicide/suicidal ideation or psychosis compared with SSZ. No effects were seen in the short or long term. Use at a higher dose or for different indications needs further investigation.

**Trial registration:**

Registered with EU PAS (reference no. EUPAS34497; http://www.encepp.eu/encepp/viewResource.htm? id=34498). The full study protocol and analysis source code can be found at https://github.com/ohdsi-studies/Covid19EstimationHydroxychloroquine2.

**Rheumatology key messages**

- This is the largest study on the neuropsychiatric safety of hydroxychloroquine, including >900 000 users internationally.
- We found no association between hydroxychloroquine treatment for RA and depression, suicide or psychosis compared with sulfasalazine.
- These findings do not support stopping hydroxychloroquine for RA based on concerns raised in COVID-19 patients.

## Introduction

Hydroxychloroquine (HCQ) has received much scientific and public attention during the coronavirus disease 2019 (COVID-19) pandemic as a leading therapeutic and prophylactic target [1, 2]. Commonly used for autoimmune disorders (e.g. SLE) and inflammatory arthritis, HCQ was released for emergency use for COVID-19 due to its postulated antiviral efficacy in cellular studies [3–9]. HCQ is currently being used in >217 registered ongoing clinical trials for the treatment of severe acute respiratory syndrome coronavirus 2 (SARS-CoV-2) as of 12 June 2020 [10, 11]. Results to date have been conflicting, with emerging data suggesting a lack of clinical efficacy against COVID-19 [12–18]. Case report literature suggests that chloroquine, the compound from which HCQ was derived, is associated with neurological and psychiatric side effects when used as an antimalarial treatment or prophylaxis [19]. Similar potential side effects that have been described in the use of HCQ include neuropsychiatric side effects such as psychosis, depression and suicidal behaviour [20–22]. Regulatory authorities have received reports of new-onset psychiatric symptoms associated with the increased use of high-dose HCQ during the pandemic [23]. While chloroquine and HCQ have multiple mechanisms of action, a major action is the disruption of lysosomal functioning and autophagy [24]. These actions to some degree mimic lysosomal storage diseases, disorders that are characterized by neurodevelopmental delay and neurodegeneration when manifested in the more common form in childhood, but also associated with neuropsychiatric manifestations in adulthood [25, 26].

New reports of serious side effects associated with HCQ used in COVID-19 are concerning to the rheumatology community, leading to confusion and anxiety for patients who are taking HCQ for autoimmune conditions. Given the previous reports of neuropsychiatric symptoms with HCQ, together with a plausible mechanism for such phenomena, we performed a review of the literature to determine what was already known about the potential risks of psychosis, depression and suicide associated with HCQ use from literature database inception until 14 May 2020 (Supplementary Appendix Section 1, available at *Rheumatology* online). Interrogation of adverse event registers have identified potential associations between HCQ and psychiatric disorders [11]. Case reports and case series describing new-onset psychosis, bipolar disorder, seizures and depression associated with HCQ and chloroquine use for rheumatologic disorders and malaria prophylaxis can be found as early as 1964 [20, 27–35]. No clinical trial or observational study was found that had investigated the incidence of new-onset neuropsychiatric symptoms associated with HCQ use.

Considering the widespread use of HCQ in rheumatology, we therefore aimed to determine whether there is an association between incident HCQ use for RA (the most common indication for the drug) and the onset of acute psychiatric events, including depression, suicide and psychosis, compared with SSZ.

## Methods

### Study design

A new-user cohort, active-comparator design was used, as recommended by methodological guidelines for observational drug safety research [36]. The study protocol is registered in the European Union Post-Authorisation Studies Register as EUPAS34497 [37].

SSZ was used as the active comparator for HCQ, as both SSZ and HCQ are second-line conventional synthetic DMARDs (csDMARDs) used in addition to or instead of MTX. While it is acknowledged that the drugs are not exactly equivalent, SSZ was felt to be the closest possible drug to HCQ in an RA cohort. Aware that there are other rheumatologic indications for using HCQ, such as SLE, we designed the study to include propensity score (PS) stratification and matching to prevent confounding. We used a set of diagnostic tools to check the PS adjustments in each dataset for any imbalances that may have remained despite stratification and also used negative control outcomes to identify if unobserved confounding had occurred. Analyses were not completed and are not reported if imbalance remained despite PS stratification or there appeared to be a large proportion of negative control outcomes outside our level of tolerance. All of these diagnostic tools were assessed while results were blinded and can be freely reviewed online. Further details are given in the statistical analysis section.

### Data sources

Electronic health records and administrative claims data from the UK and USA were used, previously mapped to the Observational Medical Outcomes Partnership (OMOP), common data model (CDM). The study period covered from September 2000 until the latest data available at the time of extraction in each database. Data from 10 data sources were analysed in a federated manner using a distributed network strategy in collaboration with the Observational Health Data Science and Informatics (OHDSI) and European Health Data and Evidence Network communities (EHDEN). The data used included primary care electronic medical records from the UK [Clinical Practice Research Datalink (CPRD) and IQVIA Medical Research Data (IMRD)]; specialist ambulatory care electronic health records from Germany [IQVIA Database Analyzer Germany (DAGermany)]; electronic health records in a sample of US inpatient and outpatient facilities in the Optum® de-identified Electronic Health Record dataset (Optum EHR) and IQVIA US Ambulatory Electronic Medical Records (AmbEMR); and US claims data from the IBM MarketScan® Commercial Claims Database (CCAE), Optum® de-identified Clinformatics® Data Mart Database–Date of Death (Clinformatics), IBM MarketScan® Medicare Supplemental Database (MDCR), IBM MarketScan® Multi-State Medicaid Database (MDCD) and IQVIA OpenClaims (OpenClaims). In addition, data were obtained and analysed from electronic primary care data from the Netherlands (IPCI database) and Spain (SIDIAP) and from Japanese claims (JMDC), but none of these analyses were deemed appropriate due to low/no event counts in at least one of the cohorts. A more detailed description of all these data sources is available in Supplementary Appendix Section 2, available at *Rheumatology* online.

### Follow-up

Participants were followed up from the date of initiation (first dispensing or prescription) of HCQ or SSZ (index date) as described in detail in Supplementary Appendix Section 3.1, available at *Rheumatology* online. SSZ was proposed as an active comparator, as it shares a similar indication as a second-line csDMARD for RA. Two different follow-up periods were prespecified to look at short- and long-term effects, respectively. First, a fixed 30 day time window from the index date was used to study short-term effects, where follow-up included from day 1 post-index until the earliest of loss to follow-up/death, outcome of interest or 30 days from therapy initiation, regardless of compliance/persistence with the study drug. Second, in a long-term (on treatment) analysis, follow-up went from day 1 post-index until the earliest of therapy discontinuation (with a 14 day additional washout), outcome of interest or loss to follow-up/death. Continued treatment episodes were constructed based on dispensing/prescription records, with a 90 day refill gap allowed to account for stockpiling.

### Participants

All subjects registered in any of the contributing data sources for at least 365 days prior to the index date, ≥18 years of age, with a history of RA (as defined by a recorded diagnosis any time before or on the same day as therapy initiation) and starting either HCQ or SSZ during the study period were included.

Potential participant counts and age-, sex- and calendar year-specific incidence per database were produced for transparency and reviewed to check for data inconsistencies and face validity and are available for inspection at https://data.ohdsi.org/Covid19CohortEvaluationExposures/, labelled as ‘New users of hydroxychloroquine with previous rheumatoid arthritis’ and ‘New users of sulfasalazine with previous rheumatoid arthritis’.

### Outcomes and confounders

Code lists for the identification of the study population, for the study exposures and for the relevant outcomes were created by clinicians with experience in the management of RA and by clinical epidemiologists using ATLAS, a science analytics platform that provides a unified interface for researchers [38]. Exposures and outcomes were reviewed by experts in Observational Medical Outcomes Partnership vocabulary and in the use of the proposed data sources. A total of three outcomes were analysed: depression, suicide or suicidal ideation and hospital admission for psychosis. Detailed outcome definitions with links to code lists are fully detailed in Supplementary Appendix Section 3.2, available at *Rheumatology* online [39, 40]. Cohort counts for each of the outcomes in the entire source database and age-, sex- and calendar year–specific incidence rates were explored for each of the contributing databases and reviewed to check for data inconsistencies and face validity. These are available for inspection at https://data.ohdsi.org/Covid19CohortEvaluationSafetyOutcomes/.

A list of negative control outcomes was generated for which there is no biologically plausible or known causal relationship with the use of HCQ or SSZ. These outcomes were identified based on previous literature, clinical knowledge (reviewed by two clinicians), product labels and spontaneous reports and confirmed by manual review by two clinicians [41]. The full list of codes used to identify negative control outcomes can be found in Supplementary Appendix Section 4, available at *Rheumatology* online.

### Statistical methods

All analytical source code is available for inspection and reproducibility at https://github.com/ohdsi-studies/Covid19EstimationHydroxychloroquine2. All study diagnostics and the steps described below are available for review at https://data.ohdsi.org/Covid19EstimationHydroxychloroquine2/.

The following steps were followed for each analysis:

#### 1. PS estimation

PS stratification was used to minimize confounding. All baseline characteristics recorded in the participants’ records/health claims were constructed for inclusion as potential confounders (including demographics, past medical history, procedures and medication prescription within 30 and within 365 days prior to drug initiation). Covariate construction details are available in Supplementary Appendix Section 5, available at *Rheumatology* online. Lasso regression models were fitted to estimate PS as the probability of HCQ *vs* SSZ use based on patient demographics and medical history, including previous conditions, procedures, healthcare resource use and treatments. The balance of known characteristics that could cause potential confounding were then reviewed while the results were blinded in order to determine whether a dataset was able to contribute to the meta-analysis. This was undertaken in two ways. First, we used the PS scores themselves and the standardized difference between the scores prior to and after PS stratification to determine whether the cohorts of SSZ and HCQ users were imbalanced. Second, we looked at the PS model pictorially in a graph to see if the populations appeared to ‘overlap’ in their characteristics. The full resulting PS models are available for inspection by clicking on ‘Propensity model’ and ‘Propensity scores’ after selecting a database in the results app (https://data.ohdsi.org/Covid19EstimationHydroxychloroquine2/).

#### 2. Study diagnostics

Study diagnostics were explored for each database-specific analysis before progressing to outcome modelling, and included checks for power, observed confounding and potential residual (unobserved) confounding. Only database-outcome analyses that passed all diagnostics below were then conducted and reported, with all others marked as ‘NA’ in the accompanying results app.

Positivity and power were assessed by looking at the number of participants in each treatment arm and the number with the outcome (see the ‘Power’ tab after clicking on a database in the results app). Small cell counts less than five (and resulting estimates) are reported as ‘<5’ to minimize the risk of secondary disclosure of data with patient identification. PS overlap was also plotted to visualize positivity issues and can be seen by clicking on ‘Propensity scores’.

Observed confounding was explored by plotting standardized differences before (*x*-axis) *vs* after (*y*-axis) PS stratification, with standardized differences >0.1 in the *y*-axis indicating the presence of unresolved confounding, which can be seen by clicking on ‘Covariate balance’ in the results app [36].

Finally, negative control outcome analyses were assessed to identify systematic error due to residual (unobserved) confounding. The results for these are available in the ‘Systematic error’ tab of the results app*.* The resulting information was used to calibrate the outcome models using empirical calibration [37, 38].

#### 3. Outcome modelling

Cox proportional hazards models conditioned on the PS strata were fitted to estimate hazard ratios (HRs) for each psychological outcome in new users of HCQ (*vs* SSZ). Empirical calibration based on the previously described negative control outcomes was used to minimize any potential residual confounding with calibrated HRs and 95% CIs estimated [42, 43]. All analyses were conducted for each database separately, with estimates combined in random-effects meta-analysis methods where *I*^2^ was ≤40% [44]. The standard errors of the database-specific estimates were adjusted to incorporate estimate variation across databases, where the across-database variance was estimated by comparing each database-specific result to that of an inverse-variance, fixed-effects meta-analysis. No meta-analysis was conducted where *I*^2^ for a given drug–outcome pair was >40%.

All analyses were conducted using the CohortMethod package, available at https://ohdsi.github.io/CohortMethod/ and the Cyclops package for PS estimation (https://ohdsi.github.io/Cyclops) [45].

##### Data sharing

Open science is a guiding principle within the OHDSI. As such, we provide unfettered access to all open-source analysis tools employed in this study via https://github.com/OHDSI/, as well as all data and results artefacts that do not include patient-level health information via http://data.ohdsi.org/Covid19EstimationHydroxychloroquine2. Data partners contributing to this study remain custodians of their individual patient-level health information and hold either institutional review board exemption or approval for participation.

## Results

A total of 918 144 HCQ and 290 383 SSZ users were identified. Participant counts in each data source are provided in Supplementary Appendix Section 6, available at *Rheumatology* online. Before PS stratification, users of HCQ were (compared with SSZ users) more likely female (e.g. 82.0% *vs* 74.3% in the CCAE database) and less likely to have certain comorbidities such as Crohn’s disease (0.6% *vs* 1.8% in the CCAE) or psoriasis (3.0% *vs* 8.9% in the CCAE). The prevalence of a past medical history of SLE was higher in HCQ users as expected (1.5% *vs* 0.5% in the CCAE), while the use of systemic glucocorticoids was similar (46.1% *vs* 47.2% in the previous month in the CCAE). The prevalence of depressive disorder was similar in both groups (13.4% *vs* 13.5% in the CCAE) and so was the history of use of antidepressants in the previous year (36.4% *vs* 36.4% in the CCAE), which appears in keeping with the prevalence discussed in previous literature [46]. After PS stratification, the prevalence of a past medical history of SLE, depressive disorder and the use of systemic glucocorticoids and antidepressants were balanced with a standard difference of <0.1 between HCQ and SSZ users. As these were balanced, these patients were not excluded from the analyses.

Detailed baseline characteristics for the two pairs of treatment groups after PS stratification in the CCAE are shown in Table 1 as an example, with the balance of SLE, depression and anti-depressant medication use included. Similar tables and a more extensive list of features are provided in Supplementary Appendix Section 7, available at *Rheumatology* online, and can also be searched for in the results app (click on a given dataset, then click on the population characteristics tab, raw and search for the condition or drug of interest). Study diagnostics including plots of propensity score distribution, covariate balance and negative control estimate distributions are provided in Supplementary Appendix Section 8.

**Table 1:** Baseline characteristics of patients with RA who are new users of HCQ *vs* SSZ, before and after PS stratification, in the CCAE database

Characteristics	Before PS stratification			After PS stratification		
	HCQ, %	SSZ, %	Std diff.	HCQ, %	SSZ, %	Std diff.
Sociodemographics						
Age group (years)						
15–19	0.6	0.6	0.00	0.6	0.6	0.00
20–24	1.9	1.9	0.00	1.8	2.0	−0.01
25–29	2.6	2.6	0.00	2.5	2.8	−0.01
30–34	4.5	4.6	0.00	4.5	4.3	0.01
35–39	7.2	7.3	0.00	7.1	7.1	0.00
40–44	9.8	9.5	0.01	9.7	9.5	0.00
45–49	13.7	12.9	0.02	13.6	13.5	0.00
50–54	18.2	18.2	0.00	18.2	18.1	0.00
55–59	20.6	21.0	−0.01	20.8	20.8	0.00
60–64	19.0	19.7	−0.02	19.4	19.8	−0.01
65–69	1.8	1.7	0.01	1.8	1.6	0.01
Gender, female	82.0	74.3	0.19	80.1	79.7	0.01
Medical history						
Acute respiratory disease	35.5	34.3	0.03	35.1	34.7	0.01
Chronic liver disease	3.2	3.2	0.00	3.2	3.4	−0.01
Chronic obstructive lung disease	4.2	4.5	−0.01	4.3	4.5	−0.01
Crohn’s disease	0.6	1.8	−0.12	0.7	1.1	−0.04
Depressive disorder	13.4	13.5	0.00	13.2	13.4	−0.01
Diabetes mellitus	13.5	13.4	0.00	13.6	13.7	0.00
Hypertensive disorder	34.7	34.9	0.00	34.7	35.0	−0.01
Obesity	9.3	9.1	0.00	9.2	9.4	−0.01
Psoriasis	3.0	8.9	−0.25	3.8	5.2	−0.07
Renal impairment	3.1	2.8	0.02	3.0	2.8	0.01
SLE	1.5	0.5	0.10	1.3	0.9	0.03
Schizophrenia	0.1	0.1	−0.01	0.1	0.1	−0.01
Ulcerative colitis	0.6	1.9	−0.12	0.7	1.0	−0.04
Medication use						
Agents acting on the renin–angiotensin system	24.3	24.9	−0.01	24.5	24.7	0.00
Antidepressants	36.4	36.4	0.00	36.3	36.5	0.00
Anti-epileptics	20.3	21.0	−0.02	20.4	20.2	0.00
Anti-inflammatory and anti-rheumatic products	55.3	57.3	−0.04	55.8	56.7	−0.02
Anti-psoriatics	0.7	1.3	−0.06	0.7	1.0	−0.03
Anti-thrombotic agents	7.4	7.3	0.01	7.4	7.3	0.00
Immunosuppressants	39.6	53.1	−0.27	43.4	43.6	0.00
Opioids	38.5	40.8	−0.05	39.0	39.3	−0.01
Psycholeptics	33.6	33.7	0.00	33.4	33.3	0.00

Std diff.: standardised difference.

The average baseline dose of HCQ was homogeneous, with >97% in the CCAE using an average dose of 420 mg daily and <3% taking an estimated dose >500 mg. All the observed differences between groups were minimized to an acceptable degree (<0.1 standardized mean differences) after PS stratification: in the CCAE, the most imbalanced variable was the use of glucocorticoids on index date, with a prevalence of 36.1% *vs* 35.8%.

Database-specific and overall counts and rates of the three study outcomes in the short- (30 day) and long-term (‘on treatment’) analyses are reported in detail in Table 2*.*Depression was the most common of the three study outcomes, with rates in the ‘on treatment’ analysis ranging from 1.99/1000 person-years among HCQ users in the CPRD to 17.74/1000 among HCQ users in the AmbEMR. Suicide/suicidal ideation was the least common outcome, with rates ranging from 0.32/1000 (HCQ users in the AmbEMR and SSZ users in the IMRD) to 14.08/1000 in SSZ users in the MDCD. Database-specific counts and incidence rates (IRs) for all three outcomes stratified by drug use are detailed in full in Supplementary Appendix Section 9, available at *Rheumatology* online.

**Fig. 1:**
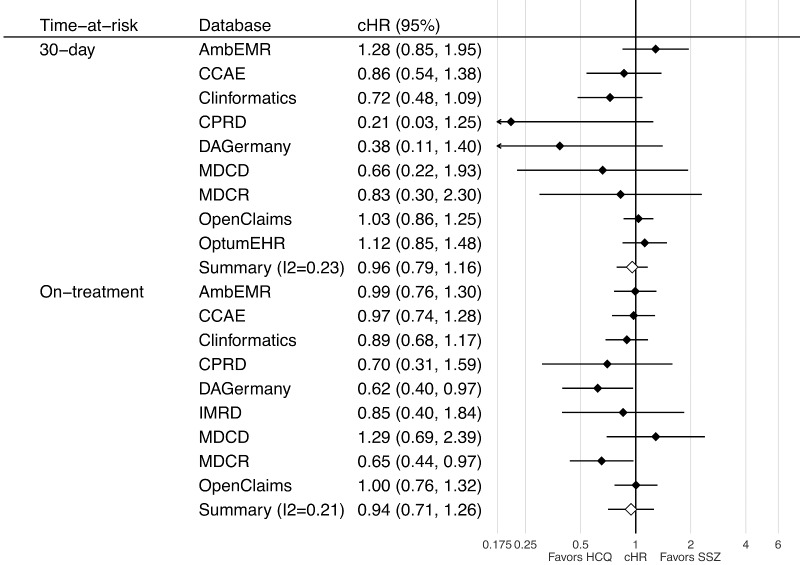
Forest plot of the association between short- (top) and long-term (bottom) use of HCQ (*vs* SSZ) and risk of depression, by database and in the meta-analysis

Nine datasets passed cohort diagnostics and contained sufficiently robust data for inclusion into the short-term analyses for depression; six passed for suicide and two passed for psychosis. A small imbalance with the incidence of a past medical history of SLE was seen in the MDCD and with cutaneous lupus in DAGermany. As a result, we excluded both from the psychosis outcome but not for depression, as we did not consider this was a confounder. Short-term (30-day) analyses showed no consistent association between HCQ use and the risk of depression, with database-specific HRs ranging from 0.21 (95% CI 0.03, 1.25) in the CPRD to 1.28 (95% CI 0.85, 1.95) in the AmbEMR, with a meta-analytic HR of 0.96 (95% CI 0.79, 1.16) (See Fig. 1, top). On-treatment analyses showed similar findings, with database-specific HRs from 0.62 (95% CI 0.40, 0.97) in DAGermany to 1.29 (95% CI 0.69, 2.39) in the MDCD, with a meta-analytic HR of 0.94 (95% CI 0.71, 1.26) (Fig. 1, bottom plot). Note only databases passing diagnostics are included within the plot and meta-analysis.

**Table 2:** Patient counts, event counts and incidence rates (IRs; per 1000 person-years) of key events according to drug use.

		30-day follow-up						On-treatment follow up					
		Patients		Events		IR (/1000 py)		Patients		Events		IR (/1000 py)	
Outcome	Database	T	C	T	C	T	C	T	C	T	C	T	C
Depression	AmbEMR	55 793	15 092	155	29	33.91	23.44	55 793	15 092	320	80	17.74	14.34
	CCAE	66 440	22 449	79	28	14.64	15.36	66 440	22 449	557	137	8.54	9.40
	Clinformatics	51 676	16 812	84	41	20.05	30.09	51 676	16 812	657	178	12.43	15.00
	CPRD	9160	11 348	<5	8	<6.67	8.60	9160	11 348	36	94	1.99	3.60
	DAGermany	3937	5109	<5	12	<15.48	28.63	3937	5109	40	70	15.47	19.66
	IMRD	8844	8456	<5	6	<6.91	8.67	8844	8456	38	51	2.20	2.72
	MDCD	7950	2286	14	6	21.61	32.29	7950	2286	90	13	15.81	10.12
	MDCR	15 735	5275	13	6	10.14	13.98	15 735	5275	97	38	5.37	9.27
	OpenClaims	620 081	183 312	654	161	12.85	10.70	620 081	183 312	4810	957	5.59	5.58
	OptumEHR	78 528	20 244	321	66	50.56	40.30	NA	NA	NA	NA	NA	NA
	Meta-analysis	918 144	290 383	<1335	363	<17.77	15.28	839 616	270 139	6645	1618	6.28	6.29
Suicide and suicidal ideation	AmbEMR	NA	NA	NA	NA	NA	NA	57 660	15 357	6	<5	0.32	<0.88
	CCAE	66 533	22 471	12	<5	2.22	<2.74	66 533	22 471	81	28	1.23	1.91
	Clinformatics	51 807	16 843	12	<5	2.85	<3.66	51 807	16 843	97	30	1.80	2.50
	CPRD	9167	11 358	<5	<5	<6.66	<5.37	9167	11 358	7	9	0.39	0.34
	IMRD	8852	8460	<5	<5	<6.91	<7.22	8852	8460	8	6	0.46	0.32
	MDCD	7980	2296	<5	<5	<7.68	<26.78	7980	2296	56	18	9.71	14.08
	MDCR	NA	NA	NA	NA	NA	NA	15 752	5278	15	6	0.83	1.45
	OpenClaims	621 067	183 550	34	8	0.67	0.53	621 067	183 550	321	89	0.37	0.52
	OptumEHR	79 903	20 480	18	8	2.78	4.82	NA	NA	NA	NA	NA	NA
	Meta-analysis	845 309	265 458	<91	<41	<1.31	<1.89	838 818	265 613	591	<191	0.55	<0.75
Hospitalization for psychosis	OpenClaims	620 964	183 527	95	27	1.86	1.79	620 964	183 527	1108	221	1.28	1.28
	OptumEHR	79 994	20 508	<5	<5	<0.77	<3.01	NA	NA	NA	NA	NA	NA
	Meta-analysis	700 958	204 035	<100	<32	<1.74	<1.91	NA	NA	NA	NA	NA	NA

T, target therapy; C, comparator therapy; IR, incidence rate; py, person-years at risk; NA, not applicable (not reported because of failed diagnostics or on-treatment follow-up unavailable); AmbEMR, IQVIA Ambulatory EMR; CCAE, IBM Commercial Database; Clinformatics, Optum de-identified Clinformatics Data Mart Database; CPRD, Clinical Practice Research Datalink; DAGermany, IQVIA Disease Analyzer Germany; IMRD, IQVIA UK Integrated Medical Record Data; MDCD, IBM Multistate Medicaid; MDCR, IBM Medicare Supplemental Database; OpenClaims, IQVIA Open Claims; OptumEHR, Optum de-identified Electronic Health Record dataset.

**Fig. 2:**
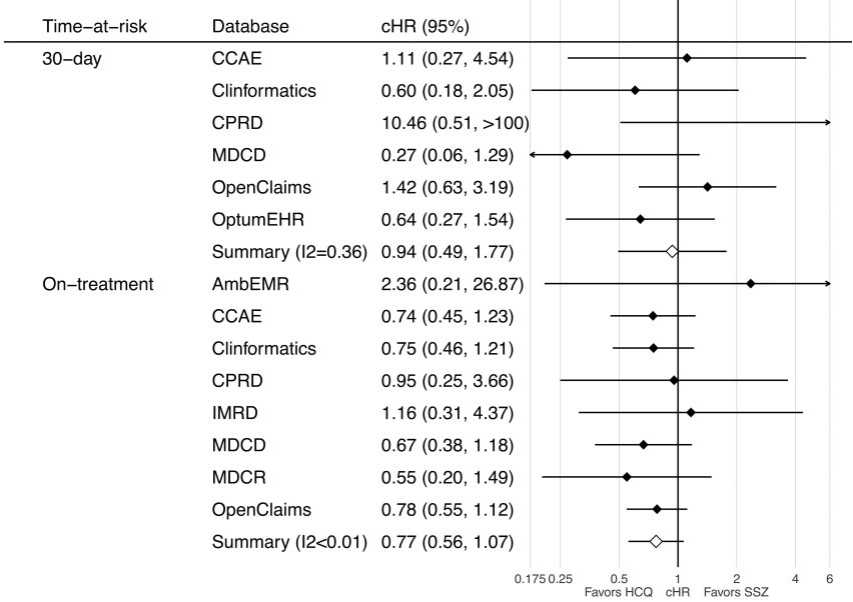
Forest plot of the association between short- (top) and long-term (bottom) use HCQ (*vs* SSZ) and risk of suicidal ideation or suicide, by database and in the meta-analysis

Similarly, no association was seen between the use of HCQ and the risk of suicidal ideation or suicide. In the short-term, HRs ranged from 0.27 (95% CI 0.06, 1.29) in the MDCD to 10.46 (95% CI 0.51, 216.29) in the CPRD, with a meta-analytic HR of 0.94 (95% CI 0.49, 1.77) (Fig. 2, top). Long-term effects were similar, with HRs ranging from 0.55 (95% CI 0.20, 1.49) in the MDCR to 2.36 (95% CI 0.21, 26.87) in the AmbEMR, with a meta-analytic HR of 0.77 (95% CI 0.56, 1.07) (Fig. 2, bottom).

Finally, no association was seen between the use of HCQ (compared with SSZ) and the risk of acute psychosis. Short-term analyses showed database-specific HRs of 0.44 (95% CI 0.05, 3.49) in the OptumEHR and 1.01 (95% CI 0.65, 1.58) in OpenClaims, with a meta-analytic estimated HR of 1.03 (95% CI 0.66, 1.60). Only OpenClaims contributed to the ‘on treatment’ analysis of this event, with an estimated HR of 0.98 (95% CI 0.73, 1.33).

## Discussion

### Principal findings

This large observational study shows that in routine healthcare treatment of RA, there is no association with the use of HCQ with acute psychosis, depression or suicide as compared with SSZ. These results are seen both in the short-term and long-term risk analyses. While an excess of psychiatric events have been reported during the COVID pandemic in those prescribed HCQ, this risk does not appear to be associated with HCQ prescribed in RA compared with those prescribed SSZ. This study uses data from three countries, with a variety of healthcare systems and modes of routine healthcare data included, enabling the study to produce more generalizable results.

### Comparison with other studies

The bulk of the evidence prior to this study consisted of isolated case reports and case series, making it difficult to draw demographic comparisons with previous work. Sato *et al.* [21] reported that neuropsychiatric adverse events found in the us Food and Drug Administration adverse event reporting system associated with chloroquine use were predominantly in females in the sixth decade of life. The increase in reporting of acute psychiatric disease during the COVID-19 pandemic may be multifactorial, with an increase in external stressors such as social isolation, financial uncertainty and increased misuse of drugs and alcohol [47–49]. Considering that we find no association for HCQ use compared with SSZ with acute psychiatric outcomes in the RA population, evidence points towards external stressors being more likely involved in the aetiology of psychiatric events seen during this pandemic.

### Strengths and weaknesses of the study

This study is based on new users of HCQ for RA and therefore the results of this study are most directly relevant to the risk of neuropsychiatric side effects seen in the rheumatologic population. The regulatory warnings of possibly increased acute psychiatric events associated with HCQ warrant investigation in all available datasets to prevent harm in both rheumatology patients and those taking it for emergency use, especially as very few clinical trials include acute psychiatric outcomes. While the general population presenting with COVID-19 may differ from those with RA, within the context of emergency authorization or off-label use of HCQ, all available evidence must be taken into account when considering the risks associated.

Several considerations must be taken into account when interpreting these results. First, the doses used to treat RA are lower than those suggested in current clinical trials for the treatment of SARS-CoV-2 and therefore adverse events seen in the treatment and prophylaxis of COVID-19 may be greater if dose dependent, as is the case with cardiac adverse effects [50, 51].

Second, this study could be affected by outcome misclassification. Only acute psychiatric events presenting to medical services will be captured, and this is especially important for the outcome of suicide. Suicide may not be fully recorded if patients do not reach medical care or cause-of-death information is not linked to the data source, and therefore the true incidence of suicide may be underrecorded [52]. Similarly, this study only focussed on acute psychosis and depression severe enough to be identified in medical consultation in patients with no history of either condition. While we generated phenotypes that underwent full cohort diagnostics, and phenotypes were constructed using a multidisciplinary team of clinicians and bioinformaticians to ensure face validity, it should be noted that no formal validation was undertaken. We took all reasonable steps to ensure the validity of the phenotypes, while considering the risk–benefit trade-off of what could be undertaken within the time frame used to respond to the serious questions raised by regulatory bodies following HCQ use in COVID-19. This study can highlight the association for patients without a prior history of psychosis or depression, but it cannot inform of the risk of acute deterioration after beginning HCQ treatment for those already known to psychiatric services.

Third, depression and hallucinations are listed as potential undesirable effects of SSZ treatment, which may underestimate the true risk, if any, from HCQ [53]. However, the frequency of depression (described as changes in affect in the summary of product characteristics for HCQ) is reported to be common (≥1/100–<1/10) while for SSZ, depression is listed as being uncommon (≥1/1000–<1/100). Therefore it is potentially reassuring for patients that we observed no difference compared with SSZ for which there is a paucity of published evidence suggesting causality [54].

PS stratification and matching as well as a comprehensive examination of potential sources of systematic error were undertaken prior to blinding of the results to identify and reduce the risk of confounding. Baseline characteristics after PS stratification were adequately balanced; of note, the incidence of SLE and a past medical history of depression and antidepressant medication use was balanced between treatment groups. Identifying the balance of these conditions between treatment groups was undertaken prior to unblinding due to the potential neuropsychiatric sequelae of the SLE aside from the potential side effects of pharmacological treatment and the increased likelihood of depression in those with a prior history. This study could also be limited by the fact that patients may overlap and exist in more than one dataset within the USA. The meta-analysis assumes populations to be independent and therefore the obtained estimates may slightly underestimate variance.

### Future research

For rheumatologic disorders, future work could expand into investigating the occurrence of acute psychiatric events in SLE patients. This would enable greater understanding of whether neuropsychiatric conditions are related to disease activity or due to pharmacological treatment. Similarly, with the emergency use of HCQ in COVID-19, there is already concern about the potential heightened risk of acute psychiatric disorders due to the increased number of psychosocial stressors present during a pandemic and high-dose use [55]. Future work should consider including acute psychiatric outcomes in order to differentiate between psychiatric conditions generated by the impact of a global pandemic compared with iatrogenic events due to the pharmaceutical therapies used.

### Meaning of the study

Exponential growth in research into the best treatment of SARS-CoV-2 infection is generating rapidly evolving evidence for the relative efficacy of pharmaceutical agents. For the rheumatologic community, media attention previously surrounded HCQ as a strong frontrunner in COVID-19 prophylaxis and treatment. The results of the RECOVERY trial, showing that dexamethasone reduced mortality in intensive care patients, have now overtaken HCQ as the leading rheumatologic drug for the pandemic, but the concerns regarding HCQ safety remain for those who take the drug for conventional indications [17, 56]. Cardiovascular safety and reports that it might lack efficacy for both treatment and prophylaxis have halted major HCQ clinical trials [50, 57–60]. The identification of acute psychiatric events associated with HCQ use has raised the need to clarify the risk within general rheumatologic use. Our study identifies no increased risk in RA patients when compared with SSZ and provides evidence to users and clinicians alike that the reports presented during the pandemic are likely to be related to further causes aside from HCQ.

## Supplementary Material

keaa771_Supplementary_Data
