# Supplementary material for: Risk of depression, suicide and psychosis with hydroxychloroquine treatment for rheumatoid arthritis: a multinational network cohort study
Source: Rheumatology (Oxford). 2020 Dec 25;60(7):3222–34. doi: 10.1093/rheumatology/keaa771 (PMC7798671; doi:10.1093/rheumatology/keaa771)
Supplement: keaa771_Supplementary_Data [file keaa771_Supplementary_Data.zip › rhe-20-2034-File004.docx]

**Supplemental Appendix to *Risk of depression, suicidal ideation, suicide and psychosis with hydroxychloroquine treatment for rheumatoid arthritis: a multi-national network cohort study***

Jennifer C.E. Lane, James Weaver, Kristin Kostka, Talita Duarte-Salles, Maria Tereza F. Abrahao, Heba Alghoul, Osaid Alser, Thamir M Alshammari, Carlos Areia, Patricia Biedermann, Juan M. Banda, Edward Burn, Paula Casajust, Kristina Fišter, Jill Hardin, Laura Hester, George Hripcsak, Benjamin Skov Kaas-Hansen, Sajan Khosla, Spyros Kolovos, Kristine E. Lynch, Rupa Makadia, Andrea V. Margulis, Michael E. Matheny, Paras P. Mehta, Daniel R. Morales, Henry Morgan-Stewart, Mees Mosseveld, Danielle Newby, Fredrik Nyberg, Anna Ostropolets, Rae Woong Park, Albert Prats-Uribe, Gowtham A. Rao, Christian Reich, Peter Rijnbeek, Anthony G. Sena, Azza Shoaibi, Matthew Spotnitz, Vignesh Subbian, Marc A. Suchard, David Vizcaya, Haini Wen, Marcel de Wilde, Junqing Xie, Seng Chan You, Lin Zhang, Simon Lovestone, Patrick Ryan, and Daniel Prieto-Alhambra

**Contents**

[1. Literature review sources 2](#_Toc45748758)

[1.1. PubMed search strategy 2](#_Toc45748759)

[1.2. EMBASE search strategy (1974 to present) 2](#_Toc45748760)

[2. Data Sources 3](#_Toc45748761)

[3. Cohort definitions 4](#_Toc45748762)

[3.1. Exposures 4](#_Toc45748763)

[3.1.1. New users of Hydroxychloroquine with prior rheumatoid arthritis 4](#_Toc45748764)

[3.1.2. New users of sulfasazine with prior rheumatoid arthritis 5](#_Toc45748765)

[3.2. Outcomes 6](#_Toc45748766)

[3.2.1. Depression 6](#_Toc45748767)

[3.2.2. Suicide and suicidal ideation 7](#_Toc45748768)

[3.2.3. Hospitalization for psychosis 7](#_Toc45748769)

[4. Negative control outcomes 8](#_Toc45748770)

[5. Covariate construction 9](#_Toc45748771)

[6. Study population counts 10](#_Toc45748772)

[7. Patient baseline characteristics before and after propensity score stratification 11](#_Toc45748773)

[7.1. AmbEMR 11](#_Toc45748774)

[7.2. CCAE 13](#_Toc45748775)

[7.3. Clinformatics 14](#_Toc45748776)

[7.4. CPRD 16](#_Toc45748777)

[7.5. DAGermany 17](#_Toc45748778)

[7.6. IMRD 19](#_Toc45748779)

[7.7. MDCD 21](#_Toc45748780)

[7.8. MDCR 23](#_Toc45748781)

[7.9. OpenClaims 24](#_Toc45748782)

[7.10. OptumEHR 26](#_Toc45748783)

[8. Study diagnostics 28](#_Toc45748784)

[8.1. Diagnostics (AmbEMR, CCAE, Clinformatics, CPRD, DAGermany) 29](#_Toc45748785)

[8.2. Diagnostics (IMRD, MDCD, MDCR, OpenClaims, OptumEHR) 30](#_Toc45748786)

[9. Incidence rates 31](#_Toc45748787)

[9.1. 30-day follow-up 31](#_Toc45748788)

[9.2. On-treatment follow-up 31](#_Toc45748789)

# Literature review sources

## PubMed search strategy

((((((((((("schizophrenia spectrum and other psychotic disorders"[mesh terms])) or (depressive disorder[mesh terms])) or (suicide, completed[mesh terms])) or (suicidal ideation[mesh terms])) or (suicide, attempted[mesh terms])) or (psychotic disorders[mesh terms])) or (psychos*[title/abstract])) or (psychot*[title/abstract])) or (depress*[title/abstract])) or (suicid*[title/abstract])) and ((((((((("hydroxychloroquine"[mesh terms]) or (hydroxychloroquine[title/abstract])) or (chloro quinol[title/abstract])) or (ercoquin[title/abstract])) or (hydroxychloroquine[title/abstract])) or (hydroycloroquine[title/abstract])) or (oxychloroquine[title/abstract])) or (plaquenil[title/abstract])) or (quensyl[title/abstract]))

## EMBASE search strategy (1974 to present)

| 1 | schizophren* ti/ab | 158752 |
| --- | --- | --- |
| 2 | depress* ti/ab | 604149 |
| 3 | psychos* ti/ab | 213154 |
| 4 | psychot* ti/ab | 134304 |
| 5 | suicid* ti/ab | 95992 |
| 6 | 1 OR 2 OR 3 OR 4 OR 5 | 1016578 |
| 7 | (Hydroxychloroquine).ti,ab | 7869 |
| 8 | (chloroquinol).ti,ab | 2 |
| 9 | (ercoquin).ti,ab | 0 |
| 10 | (hydrochloroquine).ti,ab | 57 |
| 11 | (hydrocloroquine).ti,ab | 0 |
| 12 | (oxychloroquine).ti,ab | 6 |
| 13 | (plaquenil).ti,ab | 308 |
| 14 | (quensyl).ti,ab | 4 |
| 15 | ("sn 8137").ti,ab | 1 |
| 16 | HYDROXYCHLOROQUINE/ | 23778 |
| 17 | 7 OR 8 OR 9 OR 10 OR 11 OR 12 OR 13 OR 14 OR 15 OR 16 | 24407 |
| 18 | 17 AND 6 | 451 |

# Data Sources

| **Database name** | **Abbreviation** | **Population** | **Patients (millions)** | **Data History** | **Data capture process and short database description** |
| --- | --- | --- | --- | --- | --- |
| IQVIA US Ambulatory EMR | AmbEMR | USA (General population) | 49M | 2006 – | General practice EHR, Outpatient specialist EHR - Dataset consists of longitudinal, de-identified ambulatory electronic health records data |
| IBM MarketScan Commercial Claims | CCAE | USA (Patients with commercial insurance aged <65 years) | 142M | 2000 – | Data from individuals enrolled in US employer-sponsored insurance health plans. The data includes adjudicated health insurance claims (e.g. inpatient, outpatient, and outpatient pharmacy) as well as enrollment data from large employers and health plans who provide private healthcare coverage to employees, their spouses,and dependents. Additionally, it captures laboratory tests for a subset of the covered lives. |
| Optum de-identified Clinformatics® Data Mart Database | Clinformatics | USA (Patients with commercial insurance or commercial Medicare insurance) | 85M | 2000 - | Adjudicated administrative health claims database for members of private health insurance, who are fully insured in commercial plans or in administrative services only (ASOs) and commercial Medicare. Represents inpatient and outpatient medical services and prescriptions as dispensed, as well as results for outpatient lab tests processed by national lab vendors. |
| Clinical Practice Research Datalink | CPRD | UK (General population) | 13M | 1995 – | De-identified patient data from a network of general practitioners’ practices across the UK. Primary care data are linked to a range of other health related data to provide a longitudinal, representative UK population health dataset. |
| IQVIA Disease Analyzer Germany | DAGermany | Germany (General population) | 37M | 1992 – | Anonymized patient records collected from Patient Management software used by general practitioners and selected specialists to document patients’ medical records within their office-based practice during a visit. |
| IQVIA UK Integrated Medical Record Data | IMRD | UK (General population) | 15M | 1989 – | Pseudonymized Electronic Medical Records collected from patient management software used within UK Primary Care |
| IBM MarketScan Multi-State Medicaid Database | MDCD | USA | 26M | 2006 – | Adjudicated US health insurance claims for Medicaid enrollees from multiple states and includes hospital discharge diagnoses, outpatient diagnoses and procedures, and outpatient pharmacy claims as well as ethnicity and Medicare eligibility. |
| IBM MarketScan Medicare Supplemental Database | MDCR | USA (Patients with commercial insurance aged 65+ years) | 10M | 2000 – | Represents health services of retirees (aged 65 or older) in the United States with primary or Medicare supplemental coverage through privately insured fee-for-service, point-of-service, or capitated health plans. These data include adjudicated health insurance claims (e.g. inpatient, outpatient, and outpatient pharmacy). Additionally, it captures laboratory tests for a subset of the covered lives. |
| IQVIA US LRxDx Open Claims | OpenClaims | USA (General population) | 654M | 2010 – | Pre-adjudicated claims at the anonymized patient level collected from office-based physicians and specialists via office management software and clearinghouse switch sources for the purpose of reimbursement. |
| Optum® de-identified Electronic Health Record Dataset | OptumEHR | USA (General population) | 93M | 2006 – | Optum’s de-identified electronic health record data medical records database. The medical record data includes clinical information, inclusive of prescriptions as prescribed and administered, lab results, vital signs, body measurements, diagnoses, procedures, and information derived from clinical notes using Natural Language Processing (NLP). |
|  |  |  |  |  |  |

# Cohort definitions

To view the concept sets and source code lists used in each cohort definition, navigate to the URL following the name of each cohort. Then, navigate to the Concept Sets tab. The concept sets used in defining the outcome are listed on this page. After selecting a concept set to view, a user can explore the standard concepts used in the definition or the source codes from which the standard concepts are mapped. The full specification of each cohort, including computer readable JSON and SQL representations, are publicly available at the URLs for each definition.

## Exposures

The index event was defined as the first recorded dispensing or prescription of HCQ or SSZ in a patient’s history.

Exposure commenced on the first day of dispensing or prescription recorded with at least 365 days of prior observation period to increase confidence that the exposure was incident. Exposure interval gaps of ≤90 days (HCQ and SSZ) between drug dispensing or prescription records were allowed and inferred as persistent exposure. Drug discontinuation was considered if a patient switched from exposure to the other. Patients who switched contributed follow-up time to the exposure cohort that they entered first and were censored at the time of switching in the ‘on treatment’ analysis.

### New users of Hydroxychloroquine with prior rheumatoid arthritis

<https://atlas.ohdsi.org/#/cohortdefinition/173>

Initial Event Cohort

People having any of the following:

- a drug exposure of [OHDSI Cov19] Hydroxychloroquine^1^
  - for the first time in the person's history
  - with age >= 18

with continuous observation of at least 365 days prior and 0 days after event index date, and limit initial events to: **earliest event per person.**

Inclusion Rules

Inclusion Criteria #1: has rheumatoid arthritis recorded any time on or prior to treatment

Having any of the following criteria:

- at least 1 occurrence of a condition occurrence of [OHDSI Cov19] Rheumatoid arthritis^2^

where event starts between all days Before and 0 days After index start date

- or at least 1 occurrence of an observation of [OHDSI Cov19] Rheumatoid arthritis^2^

where event starts between all days Before and 0 days After index start date

Limit qualifying cohort to: **earliest event per person.**

End Date Strategy

Custom Drug Era Exit Criteria

This strategy creates a drug era from the codes found in the specified concept set. If the index event is found within an era, the cohort end date will use the era's end date. Otherwise, it will use the observation period end date that contains the index event.

Use the era end date of [OHDSI Cov19] Hydroxychloroquine^1^

- allowing 90 days between exposures
- adding 0 days after exposure end

Cohort Collapse Strategy:

Collapse cohort by era with a gap size of 0 days.

### New users of sulfasazine with prior rheumatoid arthritis

<https://atlas.ohdsi.org/#/cohortdefinition/45>

Initial Event Cohort

People having any of the following:

- a drug exposure of [OHDSI Cov19] sulfasalazine^2^
  - for the first time in the person's history

with continuous observation of at least 365 days prior and 0 days after event index date, and limit initial events to: **earliest event per person.**

Inclusion Rules

Inclusion Criteria #1: has rheumatoid arthritis recorded any time on or prior to treatment

Having all of the following criteria:

- at least 1 occurrence of a condition occurrence of [OHDSI Cov19] Rheumatoid arthritis^1^

where event starts between all days Before and 0 days After index start date

Limit qualifying cohort to: **earliest event per person.**

End Date Strategy

Custom Drug Era Exit Criteria

This strategy creates a drug era from the codes found in the specified concept set. If the index event is found within an era, the cohort end date will use the era's end date. Otherwise, it will use the observation period end date that contains the index event.

Use the era end date of [OHDSI Cov19] sulfasalazine^2^

- allowing 90 days between exposures
- adding 0 days after exposure end

Cohort Collapse Strategy:

Collapse cohort by era with a gap size of 0 days.

## Outcomes

### Depression

<https://atlas.ohdsi.org/#/cohortdefinition/237>

Initial Event Cohort

People having any of the following:

- a condition occurrence of Depressive disorder^3^
  - for the first time in the person's history

with continuous observation of at least 365 days prior and 0 days after event index date, and limit initial events to: **earliest event per person**.

Inclusion Rules

Inclusion Criteria #1: No treatment more than 30 days prior

Having all of the following criteria:

- exactly 0 occurrences of a drug exposure of Antipsychotics^1^

where event starts between all days Before and 30 days Before index start date

- and exactly 0 occurrences of a drug exposure of SSRI^9^

where event starts between all days Before and 30 days Before index start date

- and exactly 0 occurrences of a drug exposure of SNRI^8^

where event starts between all days Before and 30 days Before index start date

- and exactly 0 occurrences of a drug exposure of Tricyclic Anti-depressants^10^

where event starts between all days Before and 30 days Before index start date

- and exactly 0 occurrences of a drug exposure of Monoamine oxidase inhibitors (MAOI), non-selective^5^

where event starts between all days Before and 30 days Before index start date

- and exactly 0 occurrences of a drug exposure of Other antidepressants^6^

where event starts between all days Before and 30 days Before index start date

Inclusion Criteria #2: No prior psychoses/mania

Having all of the following criteria:

- exactly 0 occurrences of a condition occurrence of Dementia^2^

where event starts between all days Before and 0 days After index start date

- and exactly 0 occurrences of a condition occurrence of Mania^4^

where event starts between all days Before and 0 days After index start date

- and exactly 0 occurrences of a condition occurrence of Psychosis^7^

where event starts between all days Before and 0 days After index start date

Limit qualifying cohort to: **earliest event per person**.

End Date Strategy

No end date strategy selected. By default, the cohort end date will be the end of the observation period that contains the index event.

Cohort Collapse Strategy:

Collapse cohort by era with a gap size of 0 days.

### Suicide and suicidal ideation

<https://atlas.ohdsi.org/#/cohortdefinition/235>

Initial Event Cohort

People having any of the following:

- a condition occurrence of Suicide and suicidal ideation1
- an observation of Suicide and suicidal ideation1

with continuous observation of at least 0 days prior and 0 days after event index date, and limit initial events to: **earliest event per person**.

Limit qualifying cohort to: **earliest event per person**.

End Date Strategy

No end date strategy selected. By default, the cohort end date will be the end of the observation period that contains the index event.

Cohort Collapse Strategy:

Collapse cohort by era with a gap size of 0 days.

### Hospitalization for psychosis

<https://atlas.ohdsi.org/#/cohortdefinition/236>

Initial Event Cohort

People having any of the following:

- a visit occurrence of Inpatient visit1

with continuous observation of at least 0 days prior and 0 days after event index date, and limit initial events to: **all events per person**.

For people matching the Primary Events, include:

Having all of the following criteria:

- at least 1 occurrences of a condition occurrence of Psychosis^2^
  - condition type is any of Inpatient detail - primary, Inpatient header - primary, Primary Condition, Inpatient detail - 1st position, Inpatient header - 1st position, Outpatient detail - 1st position, Outpatient header - 1st position, Carrier claim header - 1st position, Carrier claim detail - 1st position

where event starts between all days Before and 0 days After index start date and event starts between 0 days Before and all days After index end date

Limit cohort of initial events to: **all events per person**.

Limit qualifying cohort to: **all events per person**.

End Date Strategy

Date Offset Exit Criteria

This cohort definition end date will be the index event's end date plus 0 days

Cohort Collapse Strategy:

Collapse cohort by era with a gap size of 0 days.

# Negative control outcomes

Negative control outcomes are conditions believed to have no causal relationship with hydroxychloroquine, sulfasalazine, azithromycin, or amoxicillin exposure and therefore assumed *a priori* to return a hazard ratio (HR) of 1.0 when their risk is compared between exposure cohorts in a pairwise comparison. A HR of a negative control outcome that differs from 1.0 represents an estimate of the residual error of the analysis that was unaccounted for by the analytic specification such as propensity score adjustment. The distribution of residual error estimates from the negative control outcomes reflects a range of biases inherent to the analysis. The estimates on the negative control outcomes represents the empirical null distribution and we used it to compute hazard ratios and confidence intervals calibrated to reflect the observed residual error of the analysis. The negative controls were selected through a semi-automated process ^2^ and are listed below with the corresponding SNOMED concept ID.

| **Concept ID** | **Concept Name** | **Concept ID** | **Concept Name** |
| --- | --- | --- | --- |
| 378256 | Abnormal reflex | 374375 | Impacted cerumen |
| 4092879 | Absent kidney | 4344500 | Impingement syndrome of shoulder region |
| 433753 | Alcohol abuse | 440382 | Learning difficulties |
| 321689 | Apnea | 435516 | Lipoprotein deficiency disorder |
| 78200 | Benign mammary dysplasia | 438808 | Mammary duct ectasia |
| 4195873 | Breath smells unpleasant | 441553 | Myoclonus |
| 443792 | Calculus of bile duct | 4119307 | Neurogenic claudication |
| 197318 | Cholesterolosis of gallbladder | 4209423 | Nicotine dependence |
| 439125 | Complete trisomy 21 syndrome | 313601 | Oxygen supply absent |
| 433270 | Cord entanglement without compression | 4091513 | Passing flatus |
| 4311591 | Cramp in limb | 4022076 | Patient dependence on care provider |
| 441267 | Cystic fibrosis | 439971 | Poisoning by anticoagulant |
| 436233 | Delayed milestone | 46286594 | Problem related to lifestyle |
| 40486120 | Delay in physiological development | 199876 | Prolapse of female genital organs |
| 374801 | Foreign body in ear | 4049367 | Psychologic conversion disorder |
| 259995 | Foreign body in orifice | 73754 | Restless legs |
| 196456 | Gallstone | 138821 | Seborrhea |
| 4166231 | Genetic predisposition | 4198492 | Shoulder joint unstable |
| 434164 | Glycosuria | 25518 | Sickle cell trait |
| 4163735 | Hemochromatosis | 4176908 | Snapping thumb syndrome |
| 439871 | Hemospermia | 4248728 | Snoring |
| 4058388 | Hypertrophic scar | 138278 | Sprains and strains of joints and adjacent muscles |
| 435522 | Hypervitaminosis D | 4008710 | Stenosis due to any device, implant AND/OR graft |
| 443236 | Hypnotic or anxiolytic dependence | 440233 | Strain of supraspinatus muscle AND/OR tendon |
| 4098604 | Hypomagnesemia | 4194160 | Thyroid function tests abnormal |
| 435371 | Hypothermia | 4216708 | Urgent desire for stool |
| 443447 | Iatrogenic hypotension |  |  |

# Covariate construction

The following consistently extracted set of baseline patient characteristics will be constructed for input in the PS model. From this large set of typically tens of thousands of covariates, key predictors of exposure classification will be selected for inclusion in the PS model. Note that not all data sources necessarily include data for all covariates. Covariates to be included:

- Demographics (age in 5-year bands, sex, race, ethnicity, index year, index month)
- All conditions occurrence records aggregated to SNOMED clinical finding level during the following lookback windows:
  - in 365 days prior to and including index date
  - in 30 days prior to and including index date
- All drug exposure records aggregated to RxNorm ingredient level and ATC classes during the following lookback windows:
  - in 365 days prior to and including index date
  - in 30 days prior to and including index date
  - persistent exposure that overlaps index date
- All procedure occurrence records during the following lookback windows:
  - in 365 days prior to and including index date
  - in 30 days prior to and including index date
- Measurements (including laboratories) within, above, and below normal range during the following lookback window:
  - in 365 days prior to and including index date
- Device exposure records during the following lookback windows:
  - in 365 days prior to and including index date
  - in 30 days prior to and including index date
- Comorbidity or risk scores including:
  - Charlson
  - DCSI
  - CHADS2
  - CHADS2VASc

# Study population counts

The exposure cohort counts are calculated after the following study design criteria have been applied:

- Duplicate patients in the HCQ and SSZ with prior RA cohorts are removed from the cohort they qualified for second (i.e. retained in the cohort they qualified for first)
- Restricted to the calendar time when both exposures in the pairwise comparison (i.e. HCQ vs SSZ) are observed in the database.

After applying these design criteria to the original cohorts, the resulting patients are eligible to contribute data to study diagnostics and potentially to final population-level effect estimates.

| **Exposure** | **Database** | **Patients** | **Percent** |
| --- | --- | --- | --- |
| Hydroxychloroquine with prior RA (n = 923,152) | AmbEMR | 57,662 | 6.25 |
|  | CCAE | 66,656 | 7.22 |
|  | Clinformatics | 51,894 | 5.62 |
|  | CPRD | 9,169 | 0.99 |
|  | DAGermany | 3,966 | 0.43 |
|  | IMRD | 8,855 | 0.96 |
|  | MDCD | 7,994 | 0.87 |
|  | MDCR | 15,765 | 1.71 |
|  | OpenClaims | 621,124 | 67.28 |
|  | OptumEHR | 80,067 | 8.67 |
| Sulfasalazine with prior RA (n = 291,366) | AmbEMR | 15,358 | 5.27 |
|  | CCAE | 22,507 | 7.72 |
|  | Clinformatics | 16,869 | 5.79 |
|  | CPRD | 11,361 | 3.9 |
|  | DAGermany | 5,136 | 1.76 |
|  | IMRD | 8,463 | 2.9 |
|  | MDCD | 2,301 | 0.79 |
|  | MDCR | 5,281 | 1.81 |
|  | OpenClaims | 183,566 | 63 |
|  | OptumEHR | 20,524 | 7.04 |

# Patient baseline characteristics before and after propensity score stratification

## AmbEMR

|  | **Before PS stratification** | | | **After PS stratification** | | |
| --- | --- | --- | --- | --- | --- | --- |
|  | **HCQ** | **SSZ** |  | **HCQ** | **SSZ** |  |
| **Characteristic** | **%** | **%** | **Std. diff** | **%** | **%** | **Std. diff** |
| Age group |  |  |  |  |  |  |
| 15-19 | 0.1 | 0.1 | 0.00 | 0.1 | 0.1 | 0.00 |
| 20-24 | 0.7 | 0.6 | 0.01 | 0.7 | 0.6 | 0.01 |
| 25-29 | 1.3 | 1.4 | -0.01 | 1.3 | 1.4 | -0.01 |
| 30-34 | 2.4 | 2.2 | 0.02 | 2.3 | 2.2 | 0.01 |
| 35-39 | 3.5 | 3.7 | -0.01 | 3.5 | 3.4 | 0.00 |
| 40-44 | 5.4 | 5.0 | 0.02 | 5.3 | 5.1 | 0.01 |
| 45-49 | 7.4 | 7.0 | 0.02 | 7.4 | 7.4 | 0.00 |
| 50-54 | 10.4 | 11.0 | -0.02 | 10.5 | 10.4 | 0.00 |
| 55-59 | 13.1 | 13.4 | -0.01 | 13.2 | 12.9 | 0.01 |
| 60-64 | 13.8 | 14.4 | -0.02 | 13.9 | 14.1 | -0.01 |
| 65-69 | 13.7 | 14.2 | -0.01 | 13.8 | 13.7 | 0.00 |
| 70-74 | 13.1 | 13.3 | -0.01 | 13.1 | 13.9 | -0.02 |
| 75-79 | 11.2 | 9.8 | 0.05 | 10.9 | 10.9 | 0.00 |
| 80-84 | 3.9 | 3.9 | 0.00 | 3.9 | 3.7 | 0.01 |
| Gender: female | 79.7 | 74.1 | 0.13 | 78.3 | 78.4 | 0.00 |
| Race |  |  |  |  |  |  |
| race = Asian | 1.4 | 1.5 | -0.02 | 1.4 | 1.4 | 0.00 |
| race = White | 70.9 | 71.7 | -0.02 | 71.1 | 71.3 | 0.00 |
| race = African American | 8.0 | 7.8 | 0.01 | 8.0 | 7.7 | 0.01 |
| Ethnicity |  |  |  |  |  |  |
| ethnicity = Hispanic or Latino | 1.3 | 1.8 | -0.04 | 1.4 | 1.5 | -0.01 |
| ethnicity = Not Hispanic or Latino | 80.3 | 81.0 | -0.02 | 80.5 | 80.4 | 0.00 |
| Medical history: General |  |  |  |  |  |  |
| Acute respiratory disease | 13.5 | 14.2 | -0.02 | 13.7 | 13.8 | 0.00 |
| Attention deficit hyperactivity disorder | 0.6 | 0.4 | 0.02 | 0.6 | 0.5 | 0.01 |
| Chronic liver disease | 1.5 | 1.5 | 0.00 | 1.5 | 1.4 | 0.01 |
| Chronic obstructive lung disease | 7.0 | 7.2 | -0.01 | 7.1 | 6.9 | 0.01 |
| Crohn's disease | 0.4 | 1.1 | -0.08 | 0.5 | 0.6 | -0.02 |
| Dementia | 0.6 | 0.5 | 0.02 | 0.6 | 0.6 | 0.00 |
| Depressive disorder | 15.0 | 14.4 | 0.02 | 14.8 | 14.8 | 0.00 |
| Diabetes mellitus | 13.2 | 13.2 | 0.00 | 13.2 | 13.7 | -0.01 |
| Gastroesophageal reflux disease | 15.9 | 14.6 | 0.04 | 15.6 | 15.7 | 0.00 |
| Gastrointestinal hemorrhage | 1.2 | 1.2 | 0.00 | 1.2 | 1.2 | 0.00 |
| Human immunodeficiency virus infection | 0.1 | 0.1 | 0.00 | 0.1 | 0.1 | 0.00 |
| Hyperlipidemia | 29.0 | 28.1 | 0.02 | 28.8 | 29.1 | -0.01 |
| Hypertensive disorder | 38.0 | 37.1 | 0.02 | 37.8 | 38.1 | -0.01 |
| Lesion of liver | 0.9 | 0.8 | 0.01 | 0.9 | 0.8 | 0.01 |
| Lupus erythematosus | 0.9 | 0.3 | 0.08 | 0.8 | 0.7 | 0.02 |
| Obesity | 8.9 | 8.9 | 0.00 | 8.9 | 9.0 | 0.00 |
| Osteoarthritis | 25.5 | 27.1 | -0.04 | 25.8 | 25.1 | 0.02 |
| Pneumonia | 3.1 | 3.4 | -0.01 | 3.2 | 3.2 | 0.00 |
| Psoriasis | 1.5 | 3.2 | -0.11 | 1.7 | 2.0 | -0.02 |
| Renal impairment | 5.3 | 4.8 | 0.02 | 5.2 | 5.4 | -0.01 |
| Rheumatoid arthritis | 84.0 | 82.2 | 0.05 | 83.6 | 84.2 | -0.02 |
| Schizophrenia | 0.1 | 0.1 | 0.00 | 0.1 | 0.1 | 0.00 |
| Ulcerative colitis | 0.3 | 1.2 | -0.10 | 0.4 | 0.6 | -0.03 |
| Urinary tract infectious disease | 6.0 | 5.2 | 0.04 | 5.8 | 5.5 | 0.02 |
| Viral hepatitis C | 1.0 | 1.0 | 0.00 | 1.0 | 0.9 | 0.01 |
| Visual system disorder | 8.6 | 8.8 | 0.00 | 8.6 | 8.5 | 0.00 |
| Medical history: Cardiovascular disease |  |  |  |  |  |  |
| Atrial fibrillation | 3.3 | 3.1 | 0.01 | 3.2 | 3.2 | 0.00 |
| Cerebrovascular disease | 3.1 | 2.8 | 0.02 | 3.1 | 3.1 | 0.00 |
| Coronary arteriosclerosis | 5.7 | 5.6 | 0.00 | 5.7 | 5.6 | 0.00 |
| Heart disease | 15.9 | 14.6 | 0.04 | 15.6 | 15.6 | 0.00 |
| Heart failure | 2.6 | 2.3 | 0.02 | 2.6 | 2.4 | 0.01 |
| Ischemic heart disease | 1.7 | 1.6 | 0.01 | 1.7 | 1.5 | 0.02 |
| Peripheral vascular disease | 1.6 | 1.6 | 0.00 | 1.6 | 1.8 | -0.02 |
| Pulmonary embolism | 0.8 | 0.7 | 0.01 | 0.8 | 0.8 | 0.00 |
| Venous thrombosis | 1.3 | 1.2 | 0.01 | 1.3 | 1.4 | -0.01 |
| Medical history: Neoplasms |  |  |  |  |  |  |
| Hematologic neoplasm | 0.4 | 0.3 | 0.02 | 0.4 | 0.4 | 0.01 |
| Malignant lymphoma | 0.4 | 0.5 | -0.01 | 0.4 | 0.6 | -0.03 |
| Malignant neoplasm of anorectum | 0.1 | 0.1 | -0.01 | 0.1 | 0.1 | -0.01 |
| Malignant neoplastic disease | 6.9 | 6.6 | 0.01 | 6.8 | 7.0 | -0.01 |
| Malignant tumor of breast | 1.5 | 1.4 | 0.01 | 1.4 | 1.6 | -0.01 |
| Malignant tumor of colon | 0.3 | 0.3 | 0.00 | 0.3 | 0.3 | -0.01 |
| Malignant tumor of lung | 0.3 | 0.4 | -0.02 | 0.3 | 0.3 | -0.01 |
| Malignant tumor of urinary bladder | 0.2 | 0.3 | -0.01 | 0.2 | 0.2 | 0.00 |
| Primary malignant neoplasm of prostate | 0.4 | 0.3 | 0.00 | 0.4 | 0.3 | 0.00 |
| Medication use |  |  |  |  |  |  |
| Agents acting on the renin-angiotensin system | 33.2 | 33.6 | -0.01 | 33.3 | 33.6 | -0.01 |
| Antibacterials for systemic use | 24.7 | 25.3 | -0.01 | 24.9 | 24.6 | 0.01 |
| Antidepressants | 36.1 | 34.9 | 0.02 | 35.8 | 35.7 | 0.00 |
| Antiepileptics | 24.0 | 24.1 | 0.00 | 24.0 | 24.0 | 0.00 |
| Antiinflammatory and antirheumatic products | 42.8 | 44.4 | -0.03 | 43.1 | 43.5 | -0.01 |
| Antineoplastic agents | 35.9 | 41.4 | -0.11 | 37.3 | 37.6 | -0.01 |
| Antipsoriatics | 0.6 | 0.7 | 0.00 | 0.6 | 0.7 | 0.00 |
| Antithrombotic agents | 25.2 | 23.5 | 0.04 | 24.9 | 25.2 | -0.01 |
| Beta blocking agents | 25.2 | 25.1 | 0.00 | 25.2 | 25.8 | -0.02 |
| Calcium channel blockers | 17.5 | 17.7 | 0.00 | 17.5 | 17.7 | 0.00 |
| Diuretics | 30.6 | 30.0 | 0.01 | 30.5 | 31.1 | -0.01 |
| Drugs for acid related disorders | 41.6 | 41.6 | 0.00 | 41.6 | 42.1 | -0.01 |
| Drugs for obstructive airway diseases | 30.9 | 30.0 | 0.02 | 30.8 | 31.0 | 0.00 |
| Drugs used in diabetes | 14.2 | 14.3 | 0.00 | 14.3 | 14.8 | -0.01 |
| Immunosuppressants | 49.4 | 61.2 | -0.24 | 52.2 | 52.0 | 0.00 |
| Lipid modifying agents | 33.2 | 33.7 | -0.01 | 33.3 | 33.5 | 0.00 |
| Opioids | 28.4 | 30.7 | -0.05 | 28.9 | 28.8 | 0.00 |
| Psycholeptics | 26.3 | 25.6 | 0.02 | 26.1 | 26.1 | 0.00 |
| Psychostimulants, agents used for adhd and nootropics | 4.0 | 3.8 | 0.01 | 4.0 | 4.0 | 0.00 |

## CCAE

|  | **Before PS stratification** | | | **After PS stratification** | | |
| --- | --- | --- | --- | --- | --- | --- |
|  | **HCQ** | **SSZ** |  | **HCQ** | **SSZ** |  |
| **Characteristic** | **%** | **%** | **Std. diff** | **%** | **%** | **Std. diff** |
| Age group |  |  |  |  |  |  |
| 15-19 | 0.6 | 0.6 | 0.00 | 0.6 | 0.6 | 0.00 |
| 20-24 | 1.9 | 1.9 | 0.00 | 1.8 | 2.0 | -0.01 |
| 25-29 | 2.6 | 2.6 | 0.00 | 2.5 | 2.7 | -0.01 |
| 30-34 | 4.5 | 4.6 | 0.00 | 4.5 | 4.4 | 0.01 |
| 35-39 | 7.2 | 7.3 | 0.00 | 7.1 | 7.1 | 0.00 |
| 40-44 | 9.8 | 9.5 | 0.01 | 9.7 | 9.5 | 0.01 |
| 45-49 | 13.7 | 12.9 | 0.02 | 13.6 | 13.5 | 0.00 |
| 50-54 | 18.2 | 18.2 | 0.00 | 18.2 | 18.0 | 0.00 |
| 55-59 | 20.6 | 21.0 | -0.01 | 20.8 | 20.8 | 0.00 |
| 60-64 | 19.0 | 19.7 | -0.02 | 19.4 | 19.8 | -0.01 |
| 65-69 | 1.8 | 1.7 | 0.01 | 1.8 | 1.6 | 0.01 |
| Gender: female | 82.0 | 74.3 | 0.19 | 80.1 | 79.7 | 0.01 |
| Medical history: General |  |  |  |  |  |  |
| Acute respiratory disease | 35.5 | 34.3 | 0.02 | 35.1 | 34.8 | 0.01 |
| Attention deficit hyperactivity disorder | 1.5 | 1.5 | 0.00 | 1.5 | 1.5 | 0.00 |
| Chronic liver disease | 3.2 | 3.2 | 0.00 | 3.2 | 3.3 | -0.01 |
| Chronic obstructive lung disease | 4.2 | 4.5 | -0.01 | 4.3 | 4.5 | -0.01 |
| Crohn's disease | 0.6 | 1.8 | -0.12 | 0.7 | 1.1 | -0.04 |
| Dementia | 0.2 | 0.2 | 0.01 | 0.2 | 0.2 | 0.00 |
| Depressive disorder | 13.4 | 13.5 | 0.00 | 13.3 | 13.5 | -0.01 |
| Diabetes mellitus | 13.5 | 13.4 | 0.00 | 13.6 | 13.7 | -0.01 |
| Gastroesophageal reflux disease | 13.7 | 13.5 | 0.01 | 13.6 | 13.6 | 0.00 |
| Gastrointestinal hemorrhage | 2.9 | 3.4 | -0.03 | 3.0 | 3.2 | -0.01 |
| Human immunodeficiency virus infection | 0.1 | 0.2 | -0.01 | 0.1 | 0.1 | 0.00 |
| Hyperlipidemia | 31.5 | 30.6 | 0.02 | 31.2 | 31.4 | 0.00 |
| Hypertensive disorder | 34.7 | 34.9 | 0.00 | 34.7 | 35.1 | -0.01 |
| Lesion of liver | 0.9 | 0.8 | 0.01 | 0.9 | 0.8 | 0.00 |
| Lupus erythematosus | 1.5 | 0.5 | 0.10 | 1.3 | 0.9 | 0.03 |
| Obesity | 9.3 | 9.1 | 0.00 | 9.2 | 9.4 | -0.01 |
| Osteoarthritis | 43.4 | 44.3 | -0.02 | 43.5 | 44.3 | -0.01 |
| Pneumonia | 4.0 | 3.9 | 0.00 | 4.0 | 4.0 | 0.00 |
| Psoriasis | 3.0 | 8.9 | -0.25 | 3.8 | 5.2 | -0.07 |
| Renal impairment | 3.1 | 2.8 | 0.02 | 3.0 | 2.8 | 0.01 |
| Rheumatoid arthritis | 84.2 | 85.7 | -0.04 | 84.9 | 85.3 | -0.01 |
| Schizophrenia | 0.1 | 0.1 | -0.01 | 0.1 | 0.1 | -0.01 |
| Ulcerative colitis | 0.6 | 1.9 | -0.12 | 0.7 | 1.0 | -0.04 |
| Urinary tract infectious disease | 11.9 | 10.8 | 0.03 | 11.6 | 11.5 | 0.00 |
| Viral hepatitis C | 1.1 | 1.0 | 0.01 | 1.1 | 1.0 | 0.01 |
| Visual system disorder | 25.7 | 26.2 | -0.01 | 25.7 | 25.8 | 0.00 |
| Medical history: Cardiovascular disease |  |  |  |  |  |  |
| Atrial fibrillation | 1.4 | 1.3 | 0.02 | 1.4 | 1.3 | 0.01 |
| Cerebrovascular disease | 2.8 | 2.6 | 0.01 | 2.8 | 2.8 | 0.00 |
| Coronary arteriosclerosis | 4.4 | 4.6 | -0.01 | 4.4 | 4.4 | 0.00 |
| Heart disease | 15.7 | 15.0 | 0.02 | 15.5 | 15.4 | 0.00 |
| Heart failure | 1.9 | 2.0 | 0.00 | 1.9 | 1.9 | 0.00 |
| Ischemic heart disease | 2.9 | 3.2 | -0.01 | 3.0 | 3.1 | -0.01 |
| Peripheral vascular disease | 1.6 | 1.5 | 0.01 | 1.5 | 1.6 | 0.00 |
| Pulmonary embolism | 0.8 | 0.6 | 0.03 | 0.8 | 0.6 | 0.02 |
| Venous thrombosis | 1.6 | 1.4 | 0.02 | 1.5 | 1.5 | 0.00 |
| Medical history: Neoplasms |  |  |  |  |  |  |
| Hematologic neoplasm | 1.0 | 1.0 | 0.00 | 1.0 | 1.0 | 0.00 |
| Malignant lymphoma | 0.5 | 0.5 | 0.00 | 0.5 | 0.4 | 0.01 |
| Malignant neoplasm of anorectum | 0.1 | 0.1 | 0.00 | 0.1 | 0.1 | 0.00 |
| Malignant neoplastic disease | 6.5 | 6.5 | 0.00 | 6.5 | 6.5 | 0.00 |
| Malignant tumor of breast | 1.8 | 1.6 | 0.02 | 1.8 | 1.7 | 0.01 |
| Malignant tumor of colon | 0.2 | 0.2 | 0.00 | 0.2 | 0.2 | 0.00 |
| Malignant tumor of lung | 0.1 | 0.2 | -0.01 | 0.2 | 0.1 | 0.00 |
| Malignant tumor of urinary bladder | 0.1 | 0.1 | 0.00 | 0.1 | 0.1 | 0.00 |
| Primary malignant neoplasm of prostate | 0.4 | 0.4 | -0.01 | 0.4 | 0.4 | 0.00 |
| Medication use |  |  |  |  |  |  |
| Agents acting on the renin-angiotensin system | 24.3 | 24.9 | -0.01 | 24.5 | 24.7 | 0.00 |
| Antibacterials for systemic use | 43.8 | 44.3 | -0.01 | 43.8 | 43.8 | 0.00 |
| Antidepressants | 36.4 | 36.4 | 0.00 | 36.3 | 36.5 | 0.00 |
| Antiepileptics | 20.3 | 21.0 | -0.02 | 20.4 | 20.2 | 0.00 |
| Antiinflammatory and antirheumatic products | 55.3 | 57.3 | -0.04 | 55.8 | 56.8 | -0.02 |
| Antineoplastic agents | 30.7 | 37.9 | -0.15 | 33.1 | 33.1 | 0.00 |
| Antipsoriatics | 0.7 | 1.3 | -0.06 | 0.7 | 1.0 | -0.03 |
| Antithrombotic agents | 7.4 | 7.3 | 0.01 | 7.4 | 7.3 | 0.00 |
| Beta blocking agents | 15.7 | 16.2 | -0.01 | 15.9 | 16.2 | -0.01 |
| Calcium channel blockers | 11.7 | 11.5 | 0.01 | 11.7 | 11.8 | 0.00 |
| Diuretics | 24.4 | 24.3 | 0.00 | 24.5 | 24.5 | 0.00 |
| Drugs for acid related disorders | 32.3 | 33.6 | -0.03 | 32.6 | 32.6 | 0.00 |
| Drugs for obstructive airway diseases | 29.7 | 29.3 | 0.01 | 29.5 | 29.5 | 0.00 |
| Drugs used in diabetes | 10.4 | 10.5 | 0.00 | 10.5 | 10.8 | -0.01 |
| Immunosuppressants | 39.6 | 53.1 | -0.27 | 43.4 | 43.6 | 0.00 |
| Lipid modifying agents | 22.6 | 23.5 | -0.02 | 22.8 | 23.2 | -0.01 |
| Opioids | 38.5 | 40.8 | -0.05 | 39.0 | 39.3 | -0.01 |
| Psycholeptics | 33.6 | 33.7 | 0.00 | 33.4 | 33.3 | 0.00 |
| Psychostimulants, agents used for adhd and nootropics | 5.9 | 5.7 | 0.01 | 5.8 | 5.7 | 0.01 |

## Clinformatics

|  | **Before PS stratification** | | | **After PS stratification** | | |
| --- | --- | --- | --- | --- | --- | --- |
|  | **HCQ** | **SSZ** |  | **HCQ** | **SSZ** |  |
| **Characteristic** | **%** | **%** | **Std. diff** | **%** | **%** | **Std. diff** |
| Age group |  |  |  |  |  |  |
| 15-19 | 0.3 | 0.4 | -0.01 | 0.3 | 0.4 | -0.02 |
| 20-24 | 1.1 | 1.2 | -0.01 | 1.1 | 1.1 | -0.01 |
| 25-29 | 1.8 | 2.0 | -0.01 | 1.8 | 1.8 | -0.01 |
| 30-34 | 3.4 | 3.2 | 0.01 | 3.3 | 3.3 | 0.00 |
| 35-39 | 4.9 | 5.2 | -0.01 | 4.9 | 4.8 | 0.00 |
| 40-44 | 6.9 | 6.7 | 0.01 | 6.8 | 6.4 | 0.01 |
| 45-49 | 8.9 | 9.1 | 0.00 | 8.9 | 8.9 | 0.00 |
| 50-54 | 11.6 | 11.6 | 0.00 | 11.6 | 11.6 | 0.00 |
| 55-59 | 12.5 | 13.3 | -0.02 | 12.7 | 12.9 | -0.01 |
| 60-64 | 11.6 | 12.7 | -0.03 | 11.9 | 12.0 | 0.00 |
| 65-69 | 11.3 | 12.2 | -0.03 | 11.6 | 11.7 | 0.00 |
| 70-74 | 10.8 | 10.5 | 0.01 | 10.9 | 10.6 | 0.01 |
| 75-79 | 7.9 | 6.9 | 0.04 | 7.7 | 7.7 | 0.00 |
| 80-84 | 5.0 | 3.8 | 0.06 | 4.8 | 4.9 | -0.01 |
| 85-89 | 1.9 | 1.3 | 0.05 | 1.8 | 1.7 | 0.01 |
| 90-94 | 0.0 | 0.0 | 0.03 | 0.0 | 0.0 | 0.03 |
| Gender: female | 79.5 | 72.1 | 0.17 | 77.6 | 77.6 | 0.00 |
| Medical history: General |  |  |  |  |  |  |
| Acute respiratory disease | 34.6 | 33.4 | 0.02 | 34.2 | 34.2 | 0.00 |
| Attention deficit hyperactivity disorder | 1.2 | 1.1 | 0.00 | 1.2 | 1.1 | 0.00 |
| Chronic liver disease | 3.7 | 3.8 | 0.00 | 3.7 | 3.8 | -0.01 |
| Chronic obstructive lung disease | 11.2 | 10.9 | 0.01 | 11.2 | 11.2 | 0.00 |
| Crohn's disease | 0.7 | 2.4 | -0.14 | 0.9 | 1.4 | -0.05 |
| Dementia | 1.3 | 1.2 | 0.01 | 1.2 | 1.5 | -0.02 |
| Depressive disorder | 18.5 | 18.2 | 0.01 | 18.4 | 18.3 | 0.00 |
| Diabetes mellitus | 20.8 | 20.3 | 0.01 | 20.7 | 21.0 | -0.01 |
| Gastroesophageal reflux disease | 21.6 | 21.2 | 0.01 | 21.5 | 21.7 | -0.01 |
| Gastrointestinal hemorrhage | 4.0 | 4.5 | -0.02 | 4.0 | 4.0 | 0.00 |
| Human immunodeficiency virus infection | 0.2 | 0.2 | -0.01 | 0.2 | 0.2 | -0.01 |
| Hyperlipidemia | 46.7 | 45.5 | 0.02 | 46.4 | 47.1 | -0.01 |
| Hypertensive disorder | 51.3 | 51.1 | 0.00 | 51.3 | 51.7 | -0.01 |
| Lesion of liver | 1.3 | 1.2 | 0.01 | 1.3 | 1.2 | 0.00 |
| Lupus erythematosus | 1.7 | 0.7 | 0.10 | 1.5 | 1.1 | 0.03 |
| Obesity | 12.1 | 12.6 | -0.01 | 12.2 | 12.2 | 0.00 |
| Osteoarthritis | 56.3 | 55.5 | 0.02 | 56.1 | 56.7 | -0.01 |
| Pneumonia | 6.5 | 5.8 | 0.03 | 6.3 | 6.4 | 0.00 |
| Psoriasis | 3.1 | 8.1 | -0.22 | 3.8 | 4.9 | -0.05 |
| Renal impairment | 9.9 | 9.3 | 0.02 | 9.7 | 9.9 | -0.01 |
| Rheumatoid arthritis | 84.3 | 85.4 | -0.03 | 84.8 | 85.1 | -0.01 |
| Schizophrenia | 0.2 | 0.1 | 0.01 | 0.2 | 0.1 | 0.01 |
| Ulcerative colitis | 0.7 | 2.3 | -0.13 | 0.8 | 1.3 | -0.04 |
| Urinary tract infectious disease | 15.9 | 13.6 | 0.06 | 15.3 | 15.4 | 0.00 |
| Viral hepatitis C | 1.5 | 1.7 | -0.02 | 1.5 | 1.7 | -0.02 |
| Visual system disorder | 40.2 | 40.1 | 0.00 | 40.0 | 39.6 | 0.01 |
| Medical history: Cardiovascular disease |  |  |  |  |  |  |
| Atrial fibrillation | 5.0 | 4.2 | 0.04 | 4.8 | 4.7 | 0.00 |
| Cerebrovascular disease | 6.3 | 5.6 | 0.03 | 6.1 | 6.0 | 0.00 |
| Coronary arteriosclerosis | 10.8 | 10.5 | 0.01 | 10.7 | 10.9 | -0.01 |
| Heart disease | 27.7 | 26.1 | 0.04 | 27.3 | 27.8 | -0.01 |
| Heart failure | 6.3 | 5.8 | 0.02 | 6.1 | 6.4 | -0.01 |
| Ischemic heart disease | 6.5 | 6.3 | 0.01 | 6.5 | 6.6 | -0.01 |
| Peripheral vascular disease | 4.7 | 4.3 | 0.02 | 4.7 | 4.6 | 0.00 |
| Pulmonary embolism | 1.2 | 0.9 | 0.03 | 1.2 | 1.1 | 0.01 |
| Venous thrombosis | 2.3 | 2.1 | 0.02 | 2.2 | 2.4 | -0.01 |
| Medical history: Neoplasms |  |  |  |  |  |  |
| Hematologic neoplasm | 1.4 | 1.2 | 0.02 | 1.4 | 1.3 | 0.01 |
| Malignant lymphoma | 0.7 | 0.7 | -0.01 | 0.7 | 0.8 | -0.01 |
| Malignant neoplasm of anorectum | 0.2 | 0.3 | -0.02 | 0.2 | 0.2 | -0.01 |
| Malignant neoplastic disease | 10.4 | 10.4 | 0.00 | 10.4 | 10.6 | -0.01 |
| Malignant tumor of breast | 2.5 | 2.0 | 0.03 | 2.4 | 2.4 | 0.00 |
| Malignant tumor of colon | 0.4 | 0.5 | -0.01 | 0.4 | 0.5 | -0.01 |
| Malignant tumor of lung | 0.4 | 0.4 | 0.01 | 0.4 | 0.4 | 0.01 |
| Malignant tumor of urinary bladder | 0.3 | 0.4 | -0.01 | 0.3 | 0.3 | 0.00 |
| Primary malignant neoplasm of prostate | 1.0 | 1.2 | -0.02 | 1.0 | 1.0 | 0.00 |
| Medication use |  |  |  |  |  |  |
| Agents acting on the renin-angiotensin system | 32.3 | 32.7 | -0.01 | 32.4 | 32.6 | 0.00 |
| Antibacterials for systemic use | 41.2 | 42.1 | -0.02 | 41.3 | 41.2 | 0.00 |
| Antidepressants | 35.0 | 35.4 | -0.01 | 35.0 | 35.0 | 0.00 |
| Antiepileptics | 22.1 | 22.8 | -0.02 | 22.2 | 22.1 | 0.00 |
| Antiinflammatory and antirheumatic products | 48.0 | 50.0 | -0.04 | 48.5 | 49.1 | -0.01 |
| Antineoplastic agents | 30.1 | 37.9 | -0.17 | 32.6 | 32.2 | 0.01 |
| Antipsoriatics | 0.8 | 1.1 | -0.03 | 0.9 | 0.9 | 0.00 |
| Antithrombotic agents | 11.8 | 10.7 | 0.04 | 11.5 | 11.4 | 0.00 |
| Beta blocking agents | 23.2 | 22.5 | 0.02 | 23.1 | 23.1 | 0.00 |
| Calcium channel blockers | 17.8 | 16.7 | 0.03 | 17.6 | 17.7 | 0.00 |
| Diuretics | 30.3 | 29.0 | 0.03 | 30.0 | 30.5 | -0.01 |
| Drugs for acid related disorders | 33.6 | 34.6 | -0.02 | 33.8 | 34.4 | -0.01 |
| Drugs for obstructive airway diseases | 29.3 | 29.2 | 0.00 | 29.2 | 29.5 | -0.01 |
| Drugs used in diabetes | 14.1 | 14.2 | 0.00 | 14.1 | 14.5 | -0.01 |
| Immunosuppressants | 39.0 | 52.6 | -0.28 | 43.0 | 42.2 | 0.02 |
| Lipid modifying agents | 31.9 | 32.2 | -0.01 | 32.0 | 32.7 | -0.01 |
| Opioids | 38.4 | 40.2 | -0.04 | 38.8 | 38.8 | 0.00 |
| Psycholeptics | 30.4 | 29.4 | 0.02 | 30.1 | 29.9 | 0.00 |
| Psychostimulants, agents used for adhd and nootropics | 4.0 | 3.7 | 0.02 | 4.0 | 3.7 | 0.02 |

## CPRD

|  | **Before PS stratification** | | | **After PS stratification** | | |
| --- | --- | --- | --- | --- | --- | --- |
|  | **HCQ** | **SSZ** |  | **HCQ** | **SSZ** |  |
| **Characteristic** | **%** | **%** | **Std. diff** | **%** | **%** | **Std. diff** |
| Age group |  |  |  |  |  |  |
| 15-19 | 0.3 | 0.2 | 0.01 | 0.3 | 0.2 | 0.00 |
| 20-24 | 0.8 | 1.0 | -0.02 | 0.8 | 0.9 | -0.01 |
| 25-29 | 1.3 | 1.6 | -0.03 | 1.4 | 1.6 | -0.02 |
| 30-34 | 2.7 | 3.1 | -0.02 | 2.9 | 3.0 | 0.00 |
| 35-39 | 4.5 | 4.6 | 0.00 | 4.7 | 4.5 | 0.01 |
| 40-44 | 5.6 | 6.6 | -0.04 | 6.3 | 6.4 | 0.00 |
| 45-49 | 8.9 | 8.0 | 0.03 | 8.5 | 8.5 | 0.00 |
| 50-54 | 11.3 | 10.9 | 0.02 | 11.3 | 11.0 | 0.01 |
| 55-59 | 13.2 | 12.6 | 0.02 | 13.4 | 12.8 | 0.02 |
| 60-64 | 13.1 | 13.2 | 0.00 | 12.9 | 13.0 | 0.00 |
| 65-69 | 12.7 | 12.7 | 0.00 | 12.6 | 12.7 | 0.00 |
| 70-74 | 10.9 | 11.6 | -0.02 | 11.1 | 11.2 | 0.00 |
| 75-79 | 8.3 | 8.1 | 0.01 | 8.0 | 8.0 | 0.00 |
| 80-84 | 4.5 | 4.3 | 0.01 | 4.2 | 4.4 | -0.01 |
| 85-89 | 1.6 | 1.3 | 0.03 | 1.3 | 1.5 | -0.01 |
| 90-94 | 0.3 | 0.2 | 0.03 | 0.3 | 0.2 | 0.01 |
| Gender: female | 72.8 | 67.9 | 0.11 | 70.0 | 70.2 | 0.00 |
| Medical history: General |  |  |  |  |  |  |
| Acute respiratory disease | 9.2 | 9.1 | 0.00 | 9.4 | 9.3 | 0.01 |
| Chronic liver disease | 0.1 | 0.1 | 0.00 | 0.1 | 0.0 | 0.01 |
| Chronic obstructive lung disease | 2.3 | 2.0 | 0.02 | 2.2 | 2.0 | 0.01 |
| Crohn's disease | <0.1 | 0.1 | -0.03 | 0.1 | 0.1 | -0.02 |
| Dementia | 0.2 | 0.0 | 0.06 | 0.2 | 0.0 | 0.05 |
| Depressive disorder | 2.7 | 3.2 | -0.03 | 3.1 | 3.1 | 0.00 |
| Diabetes mellitus | 2.2 | 2.1 | 0.01 | 2.1 | 2.2 | 0.00 |
| Gastroesophageal reflux disease | 0.5 | 0.4 | 0.02 | 0.4 | 0.4 | 0.01 |
| Gastrointestinal hemorrhage | 1.0 | 1.4 | -0.03 | 1.1 | 1.4 | -0.02 |
| Hyperlipidemia | 1.2 | 1.0 | 0.02 | 1.2 | 1.0 | 0.01 |
| Hypertensive disorder | 3.0 | 4.0 | -0.05 | 3.2 | 3.6 | -0.02 |
| Lesion of liver | 0.1 | 0.1 | 0.01 | 0.1 | 0.1 | 0.01 |
| Lupus erythematosus | 0.2 | 0.0 | 0.05 | 0.2 | 0.0 | 0.07 |
| Obesity | 0.4 | 0.5 | -0.01 | 0.4 | 0.5 | -0.01 |
| Osteoarthritis | 7.7 | 10.4 | -0.09 | 8.6 | 8.9 | -0.01 |
| Pneumonia | 0.8 | 0.6 | 0.03 | 0.9 | 0.6 | 0.03 |
| Psoriasis | 0.8 | 1.9 | -0.10 | 1.2 | 1.5 | -0.02 |
| Renal impairment | 2.3 | 1.8 | 0.04 | 1.9 | 2.2 | -0.02 |
| Rheumatoid arthritis | 61.5 | 72.2 | -0.23 | 67.2 | 66.2 | 0.02 |
| Schizophrenia | <0.1 | 0.0 | 0.00 | 0.0 | 0.0 | 0.00 |
| Ulcerative colitis | <0.1 | 0.1 | -0.03 | 0.0 | 0.1 | -0.03 |
| Urinary tract infectious disease | 3.3 | 3.1 | 0.01 | 3.2 | 3.2 | 0.00 |
| Viral hepatitis C | <0.1 | 0.0 | 0.00 | 0.0 | 0.0 | 0.00 |
| Visual system disorder | 6.9 | 6.9 | 0.00 | 6.7 | 7.0 | -0.01 |
| Medical history: Cardiovascular disease |  |  |  |  |  |  |
| Atrial fibrillation | 0.7 | 0.7 | 0.00 | 0.8 | 0.7 | 0.02 |
| Cerebrovascular disease | 0.4 | 0.8 | -0.05 | 0.5 | 0.7 | -0.03 |
| Coronary arteriosclerosis | 0.1 | 0.1 | 0.00 | 0.1 | 0.1 | 0.00 |
| Heart disease | 3.0 | 3.3 | -0.02 | 3.3 | 3.1 | 0.02 |
| Heart failure | 0.5 | 0.7 | -0.02 | 0.7 | 0.6 | 0.01 |
| Ischemic heart disease | 1.0 | 1.5 | -0.04 | 1.2 | 1.3 | -0.01 |
| Peripheral vascular disease | 0.2 | 0.2 | 0.01 | 0.3 | 0.2 | 0.02 |
| Pulmonary embolism | 0.2 | 0.2 | -0.01 | 0.2 | 0.3 | -0.02 |
| Venous thrombosis | 0.8 | 1.0 | -0.01 | 0.9 | 1.0 | -0.01 |
| Medical history: Neoplasms |  |  |  |  |  |  |
| Hematologic neoplasm | 0.1 | 0.0 | 0.01 | 0.1 | 0.0 | 0.01 |
| Malignant lymphoma | <0.1 | 0.0 | 0.00 | 0.0 | 0.0 | 0.00 |
| Malignant neoplastic disease | 1.3 | 1.0 | 0.02 | 1.2 | 1.0 | 0.02 |
| Malignant tumor of breast | 0.1 | 0.1 | 0.00 | 0.1 | 0.1 | -0.01 |
| Malignant tumor of colon | 0.1 | 0.0 | 0.01 | 0.0 | 0.0 | 0.00 |
| Malignant tumor of lung | 0.1 | 0.0 | 0.02 | 0.1 | 0.0 | 0.02 |
| Medication use |  |  |  |  |  |  |
| Agents acting on the renin-angiotensin system | 22.1 | 16.7 | 0.14 | 19.3 | 19.3 | 0.00 |
| Antibacterials for systemic use | 37.3 | 35.0 | 0.05 | 35.9 | 36.3 | -0.01 |
| Antidepressants | 26.6 | 22.6 | 0.09 | 24.9 | 24.9 | 0.00 |
| Antiepileptics | 6.5 | 4.3 | 0.10 | 5.5 | 5.2 | 0.01 |
| Antiinflammatory and antirheumatic products | 60.5 | 67.7 | -0.15 | 64.3 | 64.0 | 0.01 |
| Antineoplastic agents | 51.5 | 28.5 | 0.48 | 39.3 | 40.8 | -0.03 |
| Antipsoriatics | 0.7 | 1.3 | -0.06 | 0.9 | 1.2 | -0.02 |
| Antithrombotic agents | 17.9 | 16.6 | 0.03 | 17.2 | 17.1 | 0.00 |
| Beta blocking agents | 14.4 | 13.4 | 0.03 | 14.3 | 13.7 | 0.02 |
| Calcium channel blockers | 15.6 | 12.0 | 0.10 | 13.4 | 13.4 | 0.00 |
| Diuretics | 19.1 | 20.8 | -0.04 | 20.0 | 20.0 | 0.00 |
| Drugs for acid related disorders | 58.3 | 51.0 | 0.15 | 54.1 | 54.6 | -0.01 |
| Drugs for obstructive airway diseases | 22.5 | 18.9 | 0.09 | 20.1 | 20.1 | 0.00 |
| Drugs used in diabetes | 7.1 | 5.8 | 0.05 | 6.1 | 6.5 | -0.02 |
| Immunosuppressants | 55.3 | 29.8 | 0.53 | 42.2 | 43.1 | -0.02 |
| Lipid modifying agents | 23.0 | 17.1 | 0.15 | 19.4 | 19.7 | -0.01 |
| Opioids | 36.3 | 41.1 | -0.10 | 39.1 | 38.8 | 0.01 |
| Psycholeptics | 16.5 | 16.4 | 0.00 | 16.6 | 16.5 | 0.00 |
| Psychostimulants, agents used for adhd and nootropics | 0.1 | 0.1 | 0.01 | 0.1 | 0.1 | 0.01 |

## DAGermany

|  | **Before PS stratification** | | | **After PS stratification** | | |
| --- | --- | --- | --- | --- | --- | --- |
|  | **HCQ** | **SSZ** |  | **HCQ** | **SSZ** |  |
| **Characteristic** | **%** | **%** | **Std. diff** | **%** | **%** | **Std. diff** |
| Age group |  |  |  |  |  |  |
| 15-19 | 0.3 | 0.5 | -0.03 | 0.3 | 0.5 | -0.04 |
| 20-24 | 1.1 | 1.3 | -0.02 | 1.1 | 1.2 | -0.01 |
| 25-29 | 1.9 | 2.4 | -0.04 | 2.1 | 2.4 | -0.02 |
| 30-34 | 2.3 | 3.6 | -0.07 | 3.0 | 3.0 | 0.00 |
| 35-39 | 4.0 | 4.1 | -0.01 | 3.9 | 4.1 | -0.01 |
| 40-44 | 5.7 | 6.6 | -0.04 | 6.0 | 6.2 | -0.01 |
| 45-49 | 8.5 | 9.2 | -0.03 | 8.9 | 9.1 | -0.01 |
| 50-54 | 12.0 | 12.6 | -0.02 | 12.7 | 12.4 | 0.01 |
| 55-59 | 14.2 | 14.8 | -0.02 | 14.6 | 15.0 | -0.01 |
| 60-64 | 13.3 | 12.1 | 0.04 | 12.3 | 12.4 | 0.00 |
| 65-69 | 11.5 | 10.7 | 0.02 | 11.3 | 10.7 | 0.02 |
| 70-74 | 10.3 | 9.5 | 0.03 | 9.8 | 9.7 | 0.00 |
| 75-79 | 9.0 | 7.4 | 0.06 | 8.2 | 8.0 | 0.00 |
| 80-84 | 4.5 | 3.9 | 0.03 | 4.3 | 4.0 | 0.02 |
| 85-89 | 1.4 | 1.0 | 0.04 | 1.3 | 1.0 | 0.03 |
| 90-94 | 0.2 | 0.2 | 0.00 | 0.2 | 0.2 | 0.00 |
| Gender: female | 81.4 | 72.7 | 0.21 | 76.2 | 77.2 | -0.02 |
| Medical history: General |  |  |  |  |  |  |
| Acute respiratory disease | 11.9 | 12.5 | -0.02 | 12.2 | 11.8 | 0.01 |
| Attention deficit hyperactivity disorder | <0.1 | 0.1 | -0.01 | 0.1 | <0.1 | 0.02 |
| Chronic liver disease | 0.6 | 0.3 | 0.05 | 0.6 | 0.3 | 0.05 |
| Chronic obstructive lung disease | 3.4 | 3.9 | -0.03 | 3.3 | 3.7 | -0.02 |
| Crohn's disease | 0.2 | 1.4 | -0.14 | 0.4 | 1.0 | -0.07 |
| Dementia | 0.6 | 0.5 | 0.01 | 0.6 | 0.5 | 0.01 |
| Depressive disorder | 7.5 | 7.7 | -0.01 | 7.6 | 7.5 | 0.01 |
| Diabetes mellitus | 6.4 | 7.1 | -0.03 | 6.9 | 6.6 | 0.01 |
| Gastroesophageal reflux disease | 1.9 | 2.0 | 0.00 | 1.8 | 1.9 | -0.01 |
| Gastrointestinal hemorrhage | 0.5 | 0.6 | -0.02 | 0.5 | 0.6 | -0.02 |
| Hyperlipidemia | 7.1 | 8.3 | -0.04 | 7.9 | 7.6 | 0.01 |
| Hypertensive disorder | 18.5 | 19.1 | -0.02 | 18.9 | 18.2 | 0.02 |
| Lesion of liver | 0.5 | 0.3 | 0.03 | 0.4 | 0.3 | 0.03 |
| Lupus erythematosus | 0.3 | <0.1 | 0.07 | 0.2 | <0.1 | 0.06 |
| Obesity | 2.2 | 2.5 | -0.02 | 2.2 | 2.4 | -0.01 |
| Osteoarthritis | 11.5 | 13.3 | -0.06 | 12.4 | 12.2 | 0.01 |
| Pneumonia | 1.9 | 1.4 | 0.04 | 2.0 | 1.5 | 0.03 |
| Psoriasis | 2.3 | 4.5 | -0.12 | 3.6 | 3.5 | 0.00 |
| Renal impairment | 2.7 | 2.2 | 0.03 | 2.5 | 2.4 | 0.01 |
| Rheumatoid arthritis | 46.1 | 54.6 | -0.17 | 52.1 | 49.9 | 0.04 |
| Schizophrenia | <0.1 | 0.1 | -0.03 | <0.1 | 0.1 | -0.01 |
| Ulcerative colitis | 0.2 | 1.6 | -0.16 | 0.4 | 1.1 | -0.09 |
| Urinary tract infectious disease | 4.0 | 4.0 | 0.00 | 4.0 | 3.9 | 0.00 |
| Viral hepatitis C | 0.3 | 0.1 | 0.04 | 0.3 | 0.1 | 0.04 |
| Visual system disorder | 5.7 | 5.5 | 0.01 | 5.4 | 5.4 | 0.00 |
| Medical history: Cardiovascular disease |  |  |  |  |  |  |
| Atrial fibrillation | 0.8 | 0.8 | 0.00 | 0.8 | 0.8 | 0.00 |
| Cerebrovascular disease | 1.4 | 1.7 | -0.03 | 1.4 | 1.6 | -0.02 |
| Coronary arteriosclerosis | 1.0 | 1.1 | -0.01 | 1.0 | 1.2 | -0.02 |
| Heart disease | 10.5 | 11.4 | -0.03 | 10.5 | 10.7 | 0.00 |
| Heart failure | 2.0 | 2.6 | -0.04 | 2.0 | 2.4 | -0.03 |
| Ischemic heart disease | 4.4 | 5.1 | -0.04 | 4.6 | 4.5 | 0.00 |
| Peripheral vascular disease | 1.3 | 1.0 | 0.02 | 1.2 | 0.9 | 0.02 |
| Pulmonary embolism | 0.6 | 0.3 | 0.04 | 0.6 | 0.4 | 0.03 |
| Venous thrombosis | 1.2 | 1.4 | -0.02 | 1.4 | 1.5 | 0.00 |
| Medical history: Neoplasms |  |  |  |  |  |  |
| Hematologic neoplasm | 0.3 | 0.2 | 0.03 | 0.3 | 0.2 | 0.02 |
| Malignant lymphoma | 0.2 | 0.2 | 0.01 | 0.2 | 0.1 | 0.01 |
| Malignant neoplasm of anorectum | 0.2 | 0.2 | 0.02 | 0.2 | 0.2 | 0.00 |
| Malignant neoplastic disease | 3.3 | 3.2 | 0.00 | 3.2 | 3.2 | 0.00 |
| Malignant tumor of breast | 0.7 | 0.6 | 0.00 | 0.6 | 0.6 | 0.00 |
| Malignant tumor of colon | 0.3 | 0.1 | 0.03 | 0.3 | 0.1 | 0.04 |
| Malignant tumor of lung | <0.1 | <0.1 | 0.02 | <0.1 | <0.1 | 0.00 |
| Malignant tumor of urinary bladder | <0.1 | 0.2 | -0.05 | <0.1 | 0.2 | -0.05 |
| Primary malignant neoplasm of prostate | 0.2 | 0.3 | -0.03 | 0.2 | 0.3 | -0.02 |
| Medication use |  |  |  |  |  |  |
| Agents acting on the renin-angiotensin system | 16.7 | 17.0 | -0.01 | 16.9 | 16.5 | 0.01 |
| Antibacterials for systemic use | 12.7 | 14.6 | -0.06 | 13.4 | 13.6 | -0.01 |
| Antidepressants | 8.8 | 7.7 | 0.04 | 8.1 | 8.0 | 0.00 |
| Antiepileptics | 3.0 | 2.3 | 0.05 | 2.6 | 2.3 | 0.02 |
| Antiinflammatory and antirheumatic products | 33.1 | 35.9 | -0.06 | 35.3 | 34.6 | 0.02 |
| Antineoplastic agents | 31.5 | 26.7 | 0.10 | 29.2 | 30.3 | -0.02 |
| Antipsoriatics | 0.3 | 0.3 | 0.00 | 0.3 | 0.2 | 0.00 |
| Antithrombotic agents | 10.0 | 9.9 | 0.00 | 9.6 | 9.3 | 0.01 |
| Beta blocking agents | 13.7 | 13.2 | 0.02 | 12.8 | 13.0 | 0.00 |
| Calcium channel blockers | 7.5 | 7.7 | -0.01 | 7.4 | 7.3 | 0.00 |
| Diuretics | 13.9 | 14.5 | -0.02 | 14.3 | 13.6 | 0.02 |
| Drugs for acid related disorders | 32.0 | 32.5 | -0.01 | 32.1 | 32.4 | -0.01 |
| Drugs for obstructive airway diseases | 6.9 | 7.7 | -0.03 | 7.2 | 7.2 | 0.00 |
| Drugs used in diabetes | 4.4 | 4.8 | -0.02 | 4.7 | 4.6 | 0.01 |
| Immunosuppressants | 40.3 | 35.0 | 0.11 | 38.7 | 38.4 | 0.00 |
| Lipid modifying agents | 7.3 | 9.1 | -0.06 | 8.0 | 8.1 | 0.00 |
| Opioids | 12.3 | 12.9 | -0.02 | 12.2 | 12.2 | 0.00 |
| Psycholeptics | 4.8 | 5.9 | -0.05 | 4.8 | 5.3 | -0.02 |
| Psychostimulants, agents used for adhd and nootropics | 0.2 | 0.4 | -0.03 | 0.2 | 0.3 | -0.03 |

## IMRD

|  | **Before PS stratification** | | | **After PS stratification** | | |
| --- | --- | --- | --- | --- | --- | --- |
|  | **HCQ** | **SSZ** |  | **HCQ** | **SSZ** |  |
| **Characteristic** | **%** | **%** | **Std. diff** | **%** | **%** | **Std. diff** |
| Age group |  |  |  |  |  |  |
| 15-19 | 0.2 | 0.2 | 0.02 | 0.2 | 0.2 | 0.01 |
| 20-24 | 0.8 | 1.0 | -0.03 | 0.8 | 1.0 | -0.02 |
| 25-29 | 1.2 | 1.6 | -0.03 | 1.4 | 1.6 | -0.02 |
| 30-34 | 2.8 | 3.2 | -0.02 | 2.9 | 3.1 | -0.01 |
| 35-39 | 4.8 | 5.0 | -0.01 | 5.0 | 4.8 | 0.01 |
| 40-44 | 6.0 | 6.1 | 0.00 | 6.3 | 6.0 | 0.01 |
| 45-49 | 8.7 | 7.8 | 0.03 | 8.4 | 8.5 | 0.00 |
| 50-54 | 11.3 | 10.8 | 0.02 | 11.2 | 11.0 | 0.01 |
| 55-59 | 13.0 | 12.4 | 0.02 | 12.7 | 12.4 | 0.01 |
| 60-64 | 13.2 | 13.8 | -0.02 | 13.4 | 13.5 | 0.00 |
| 65-69 | 13.0 | 12.8 | 0.01 | 12.9 | 13.0 | 0.00 |
| 70-74 | 11.0 | 11.4 | -0.01 | 11.3 | 11.2 | 0.00 |
| 75-79 | 7.7 | 8.3 | -0.02 | 7.8 | 7.9 | 0.00 |
| 80-84 | 4.4 | 4.1 | 0.02 | 4.1 | 4.2 | 0.00 |
| 85-89 | 1.5 | 1.3 | 0.02 | 1.3 | 1.4 | -0.01 |
| 90-94 | 0.3 | 0.2 | 0.02 | 0.2 | 0.2 | 0.00 |
| Gender: female | 73.2 | 68.0 | 0.11 | 70.7 | 71.0 | 0.00 |
| Race |  |  |  |  |  |  |
| race = Asian | 0.1 | 0.1 | 0.02 | 0.1 | 0.1 | 0.00 |
| race = White | 29.8 | 25.8 | 0.09 | 27.4 | 27.6 | 0.00 |
| race = Asian Indian | 0.7 | 0.4 | 0.04 | 0.6 | 0.5 | 0.02 |
| race = Bangladeshi | 0.1 | <0.1 | 0.02 | <0.1 | <0.1 | 0.02 |
| race = Chinese | 0.1 | <0.1 | 0.03 | 0.1 | <0.1 | 0.03 |
| race = Pakistani | 0.3 | 0.2 | 0.03 | 0.3 | 0.2 | 0.03 |
| race = Black | 0.4 | 0.2 | 0.03 | 0.3 | 0.2 | 0.02 |
| race = African | 0.2 | <0.1 | 0.05 | 0.2 | <0.1 | 0.04 |
| race = European | 0.1 | <0.1 | 0.02 | 0.1 | <0.1 | 0.02 |
| Medical history: General |  |  |  |  |  |  |
| Acute respiratory disease | 9.2 | 9.3 | 0.00 | 9.2 | 9.1 | 0.00 |
| Chronic liver disease | 0.1 | 0.1 | 0.00 | <0.1 | <0.1 | 0.00 |
| Chronic obstructive lung disease | 2.1 | 2.5 | -0.02 | 2.2 | 2.3 | -0.01 |
| Crohn's disease | <0.1 | 0.2 | -0.05 | <0.1 | 0.1 | -0.04 |
| Dementia | 0.2 | 0.1 | 0.03 | 0.1 | 0.1 | 0.02 |
| Depressive disorder | 2.6 | 2.9 | -0.02 | 2.9 | 2.7 | 0.01 |
| Diabetes mellitus | 2.3 | 2.2 | 0.01 | 2.2 | 2.1 | 0.01 |
| Gastroesophageal reflux disease | 0.3 | 0.6 | -0.03 | 0.4 | 0.6 | -0.03 |
| Gastrointestinal hemorrhage | 0.9 | 1.3 | -0.04 | 1.0 | 1.1 | -0.01 |
| Hyperlipidemia | 1.1 | 1.3 | -0.02 | 1.1 | 1.2 | -0.01 |
| Hypertensive disorder | 3.1 | 3.7 | -0.03 | 3.2 | 3.2 | 0.00 |
| Lesion of liver | 0.1 | 0.1 | 0.01 | 0.1 | <0.1 | 0.01 |
| Lupus erythematosus | 0.1 | 0.0 |  | 0.1 | 0.0 |  |
| Obesity | 0.3 | 0.5 | -0.03 | 0.4 | 0.6 | -0.03 |
| Osteoarthritis | 7.5 | 9.4 | -0.07 | 8.1 | 8.1 | 0.00 |
| Pneumonia | 0.7 | 0.6 | 0.00 | 0.7 | 0.7 | 0.00 |
| Psoriasis | 0.8 | 2.2 | -0.12 | 1.3 | 1.6 | -0.02 |
| Renal impairment | 2.5 | 2.2 | 0.01 | 2.3 | 2.3 | 0.00 |
| Rheumatoid arthritis | 61.4 | 69.3 | -0.17 | 65.7 | 63.7 | 0.04 |
| Schizophrenia | <0.1 | <0.1 | 0.01 | <0.1 | <0.1 | 0.02 |
| Ulcerative colitis | <0.1 | <0.1 | -0.01 | <0.1 | <0.1 | -0.01 |
| Urinary tract infectious disease | 3.1 | 2.9 | 0.01 | 3.1 | 3.0 | 0.01 |
| Visual system disorder | 6.6 | 7.0 | -0.02 | 6.6 | 6.8 | -0.01 |
| Medical history: Cardiovascular disease |  |  |  |  |  |  |
| Atrial fibrillation | 0.7 | 0.7 | 0.00 | 0.7 | 0.6 | 0.01 |
| Cerebrovascular disease | 0.5 | 0.7 | -0.03 | 0.5 | 0.7 | -0.02 |
| Coronary arteriosclerosis | 0.1 | 0.1 | 0.01 | 0.1 | 0.1 | 0.02 |
| Heart disease | 2.7 | 2.9 | -0.01 | 2.8 | 2.6 | 0.02 |
| Heart failure | 0.4 | 0.4 | -0.01 | 0.5 | 0.4 | 0.01 |
| Ischemic heart disease | 0.8 | 1.2 | -0.04 | 1.0 | 1.0 | 0.00 |
| Peripheral vascular disease | 0.2 | 0.2 | 0.01 | 0.3 | 0.2 | 0.02 |
| Pulmonary embolism | 0.2 | 0.2 | -0.01 | 0.2 | 0.3 | -0.02 |
| Venous thrombosis | 0.8 | 1.1 | -0.03 | 0.8 | 1.0 | -0.02 |
| Medical history: Neoplasms |  |  |  |  |  |  |
| Hematologic neoplasm | 0.1 | 0.1 | 0.01 | 0.1 | <0.1 | 0.02 |
| Malignant lymphoma | <0.1 | <0.1 | -0.01 | <0.1 | <0.1 | 0.00 |
| Malignant neoplasm of anorectum | <0.1 | <0.1 | 0.02 | <0.1 | <0.1 | 0.02 |
| Malignant neoplastic disease | 1.3 | 1.1 | 0.02 | 1.2 | 1.0 | 0.02 |
| Malignant tumor of breast | 0.2 | 0.2 | 0.02 | 0.2 | 0.2 | 0.01 |
| Malignant tumor of colon | <0.1 | <0.1 | -0.01 | <0.1 | <0.1 | 0.00 |
| Malignant tumor of lung | 0.1 | 0.1 | 0.01 | 0.1 | 0.1 | 0.02 |
| Medication use |  |  |  |  |  |  |
| Agents acting on the renin-angiotensin system | 0.6 | 0.6 | 0.00 | 0.6 | 0.8 | -0.02 |
| Antibacterials for systemic use | 3.8 | 3.4 | 0.02 | 3.6 | 3.5 | 0.01 |
| Antidepressants | 1.1 | 1.1 | -0.01 | 1.0 | 1.2 | -0.02 |
| Antiepileptics | 0.1 | 0.2 | -0.03 | 0.1 | 0.2 | -0.03 |
| Antiinflammatory and antirheumatic products | 1.6 | 1.9 | -0.03 | 1.7 | 1.7 | -0.01 |
| Antineoplastic agents | 1.5 | 0.6 | 0.08 | 1.2 | 0.9 | 0.02 |
| Antithrombotic agents | 1.1 | 1.1 | 0.00 | 1.2 | 1.2 | 0.00 |
| Beta blocking agents | 0.4 | 0.6 | -0.02 | 0.5 | 0.6 | -0.01 |
| Calcium channel blockers | 0.6 | 0.5 | 0.00 | 0.6 | 0.5 | 0.02 |
| Diuretics | 0.8 | 1.0 | -0.02 | 1.0 | 0.9 | 0.01 |
| Drugs for acid related disorders | 1.6 | 1.7 | 0.00 | 1.7 | 1.6 | 0.01 |
| Drugs for obstructive airway diseases | 0.4 | 0.4 | 0.00 | 0.5 | 0.4 | 0.00 |
| Drugs used in diabetes | 0.2 | 0.2 | 0.01 | 0.2 | 0.2 | 0.00 |
| Immunosuppressants | 1.6 | 0.7 | 0.08 | 1.3 | 1.0 | 0.02 |
| Lipid modifying agents | 0.9 | 0.9 | 0.00 | 0.9 | 1.0 | -0.01 |
| Opioids | 0.6 | 0.6 | -0.01 | 0.6 | 0.6 | -0.01 |
| Psycholeptics | 0.4 | 0.5 | -0.01 | 0.4 | 0.4 | 0.00 |

## MDCD

|  | **Before PS stratification** | | | **After PS stratification** | | |
| --- | --- | --- | --- | --- | --- | --- |
|  | **HCQ** | **SSZ** |  | **HCQ** | **SSZ** |  |
| **Characteristic** | **%** | **%** | **Std. diff** | **%** | **%** | **Std. diff** |
| Age group |  |  |  |  |  |  |
| 15-19 | 1.2 | 1.3 | -0.01 | 1.2 | 1.5 | -0.02 |
| 20-24 | 2.2 | 2.7 | -0.04 | 2.1 | 3.3 | -0.07 |
| 25-29 | 4.3 | 4.2 | 0.01 | 4.2 | 4.6 | -0.02 |
| 30-34 | 6.7 | 6.6 | 0.00 | 6.5 | 6.6 | 0.00 |
| 35-39 | 9.0 | 8.8 | 0.01 | 8.7 | 9.0 | -0.01 |
| 40-44 | 10.2 | 9.1 | 0.04 | 10.1 | 9.0 | 0.04 |
| 45-49 | 13.3 | 12.8 | 0.02 | 13.3 | 13.7 | -0.01 |
| 50-54 | 15.9 | 16.2 | -0.01 | 16.2 | 15.8 | 0.01 |
| 55-59 | 15.7 | 16.3 | -0.01 | 16.0 | 15.2 | 0.02 |
| 60-64 | 12.0 | 13.7 | -0.05 | 12.2 | 12.9 | -0.02 |
| 65-69 | 4.5 | 4.0 | 0.02 | 4.6 | 4.1 | 0.02 |
| 70-74 | 2.0 | 2.0 | -0.01 | 2.0 | 1.8 | 0.01 |
| 75-79 | 1.6 | 1.0 | 0.05 | 1.6 | 1.1 | 0.05 |
| 80-84 | 1.0 | 0.9 | 0.01 | 1.0 | 0.9 | 0.01 |
| 85-89 | 0.4 | 0.2 | 0.03 | 0.4 | 0.3 | 0.00 |
| Gender: female | 86.0 | 79.1 | 0.18 | 84.4 | 84.3 | 0.00 |
| Race |  |  |  |  |  |  |
| race = Black or African American | 28.7 | 25.2 | 0.08 | 28.0 | 26.3 | 0.04 |
| race = White | 54.4 | 57.1 | -0.05 | 55.1 | 55.3 | -0.01 |
| Ethnicity |  |  |  |  |  |  |
| ethnicity = Hispanic or Latino | 2.4 | 1.9 | 0.03 | 2.4 | 2.1 | 0.02 |
| Medical history: General |  |  |  |  |  |  |
| Acute respiratory disease | 41.9 | 40.2 | 0.03 | 41.6 | 41.4 | 0.00 |
| Attention deficit hyperactivity disorder | 2.6 | 2.0 | 0.04 | 2.6 | 2.5 | 0.00 |
| Chronic liver disease | 6.9 | 7.4 | -0.02 | 6.9 | 7.1 | -0.01 |
| Chronic obstructive lung disease | 21.1 | 21.5 | -0.01 | 21.2 | 21.0 | 0.00 |
| Crohn's disease | 0.8 | 3.4 | -0.18 | 1.0 | 1.8 | -0.07 |
| Dementia | 1.2 | 0.9 | 0.03 | 1.1 | 1.1 | 0.00 |
| Depressive disorder | 36.0 | 35.3 | 0.02 | 35.4 | 36.2 | -0.02 |
| Diabetes mellitus | 25.5 | 25.6 | 0.00 | 25.5 | 25.5 | 0.00 |
| Gastroesophageal reflux disease | 29.6 | 30.4 | -0.02 | 29.5 | 30.4 | -0.02 |
| Gastrointestinal hemorrhage | 5.0 | 6.3 | -0.06 | 5.0 | 6.0 | -0.04 |
| Human immunodeficiency virus infection | 0.5 | 0.8 | -0.04 | 0.5 | 0.8 | -0.04 |
| Hyperlipidemia | 36.4 | 36.9 | -0.01 | 36.3 | 37.7 | -0.03 |
| Hypertensive disorder | 55.6 | 53.3 | 0.05 | 55.2 | 54.3 | 0.02 |
| Lesion of liver | 2.0 | 2.0 | 0.00 | 2.0 | 2.0 | 0.00 |
| Lupus erythematosus | 3.0 | 1.2 | 0.13 | 2.8 | 1.8 | 0.07 |
| Obesity | 21.4 | 20.3 | 0.03 | 21.2 | 20.6 | 0.01 |
| Osteoarthritis | 57.2 | 56.7 | 0.01 | 57.3 | 57.4 | 0.00 |
| Pneumonia | 8.7 | 8.1 | 0.02 | 8.4 | 7.9 | 0.02 |
| Psoriasis | 2.0 | 6.4 | -0.22 | 2.5 | 3.4 | -0.05 |
| Renal impairment | 9.4 | 8.0 | 0.05 | 9.2 | 8.3 | 0.03 |
| Rheumatoid arthritis | 80.7 | 84.9 | -0.11 | 81.9 | 83.2 | -0.03 |
| Schizophrenia | 1.7 | 2.0 | -0.02 | 1.6 | 2.1 | -0.03 |
| Ulcerative colitis | 0.5 | 1.6 | -0.11 | 0.6 | 1.3 | -0.07 |
| Urinary tract infectious disease | 17.8 | 16.6 | 0.03 | 17.3 | 16.7 | 0.02 |
| Viral hepatitis C | 5.9 | 6.0 | -0.01 | 5.9 | 6.2 | -0.02 |
| Visual system disorder | 40.0 | 40.5 | -0.01 | 40.0 | 39.5 | 0.01 |
| Medical history: Cardiovascular disease |  |  |  |  |  |  |
| Atrial fibrillation | 3.3 | 2.5 | 0.05 | 3.2 | 3.2 | 0.00 |
| Cerebrovascular disease | 5.3 | 5.3 | 0.00 | 5.1 | 5.6 | -0.02 |
| Coronary arteriosclerosis | 9.8 | 8.6 | 0.04 | 9.6 | 9.4 | 0.01 |
| Heart disease | 29.6 | 27.2 | 0.05 | 29.0 | 27.9 | 0.02 |
| Heart failure | 8.7 | 7.5 | 0.04 | 8.4 | 8.0 | 0.01 |
| Ischemic heart disease | 7.3 | 7.0 | 0.01 | 7.1 | 7.4 | -0.01 |
| Peripheral vascular disease | 4.8 | 4.2 | 0.03 | 4.6 | 4.2 | 0.02 |
| Pulmonary embolism | 2.0 | 1.1 | 0.08 | 1.9 | 1.6 | 0.02 |
| Venous thrombosis | 3.1 | 2.5 | 0.03 | 2.9 | 3.3 | -0.02 |
| Medical history: Neoplasms |  |  |  |  |  |  |
| Hematologic neoplasm | 1.5 | 1.2 | 0.03 | 1.4 | 1.2 | 0.02 |
| Malignant lymphoma | 0.4 | 0.5 | -0.01 | 0.4 | 0.4 | 0.01 |
| Malignant neoplasm of anorectum | 0.2 | 0.2 | -0.01 | 0.2 | <0.2 | 0.00 |
| Malignant neoplastic disease | 7.2 | 7.5 | -0.01 | 7.0 | 7.3 | -0.01 |
| Malignant tumor of breast | 2.0 | 1.7 | 0.02 | 1.9 | 1.5 | 0.03 |
| Malignant tumor of colon | 0.4 | 0.3 | 0.01 | 0.4 | 0.3 | 0.02 |
| Malignant tumor of lung | 0.4 | 0.3 | 0.03 | 0.4 | 0.3 | 0.01 |
| Malignant tumor of urinary bladder | 0.2 | <0.2 | 0.01 | 0.2 | <0.2 | 0.02 |
| Primary malignant neoplasm of prostate | 0.2 | 0.2 | -0.01 | 0.2 | <0.2 | 0.01 |
| Medication use |  |  |  |  |  |  |
| Agents acting on the renin-angiotensin system | 32.6 | 32.2 | 0.01 | 32.6 | 33.2 | -0.01 |
| Antibacterials for systemic use | 50.4 | 48.6 | 0.04 | 50.1 | 48.0 | 0.04 |
| Antidepressants | 53.7 | 52.7 | 0.02 | 53.5 | 53.2 | 0.00 |
| Antiepileptics | 43.0 | 41.6 | 0.03 | 42.6 | 42.7 | 0.00 |
| Antiinflammatory and antirheumatic products | 58.3 | 59.8 | -0.03 | 58.4 | 59.7 | -0.03 |
| Antineoplastic agents | 30.3 | 42.0 | -0.25 | 34.1 | 31.5 | 0.05 |
| Antipsoriatics | 0.8 | 0.7 | 0.02 | 0.8 | 0.6 | 0.03 |
| Antithrombotic agents | 18.8 | 17.0 | 0.04 | 18.5 | 17.7 | 0.02 |
| Beta blocking agents | 24.9 | 22.8 | 0.05 | 24.5 | 23.0 | 0.04 |
| Calcium channel blockers | 20.0 | 19.5 | 0.01 | 19.6 | 20.3 | -0.02 |
| Diuretics | 33.7 | 32.3 | 0.03 | 33.3 | 32.0 | 0.03 |
| Drugs for acid related disorders | 50.1 | 50.7 | -0.01 | 50.3 | 48.1 | 0.04 |
| Drugs for obstructive airway diseases | 46.3 | 46.5 | 0.00 | 46.1 | 46.7 | -0.01 |
| Drugs used in diabetes | 18.4 | 19.8 | -0.03 | 18.5 | 19.4 | -0.02 |
| Immunosuppressants | 39.5 | 56.5 | -0.35 | 44.5 | 40.8 | 0.07 |
| Lipid modifying agents | 29.1 | 30.1 | -0.02 | 29.2 | 30.6 | -0.03 |
| Opioids | 62.1 | 62.5 | -0.01 | 62.0 | 62.7 | -0.02 |
| Psycholeptics | 49.8 | 49.5 | 0.00 | 49.3 | 50.2 | -0.02 |
| Psychostimulants, agents used for adhd and nootropics | 7.3 | 6.8 | 0.02 | 7.2 | 7.4 | -0.01 |

## MDCR

|  | **Before PS stratification** | | | **After PS stratification** | | |
| --- | --- | --- | --- | --- | --- | --- |
|  | **HCQ** | **SSZ** |  | **HCQ** | **SSZ** |  |
| **Characteristic** | **%** | **%** | **Std. diff** | **%** | **%** | **Std. diff** |
| Age group |  |  |  |  |  |  |
| 45-49 | 0.1 | <0.1 | 0.01 | 0.1 | <0.1 | 0.01 |
| 50-54 | 0.3 | 0.3 | -0.01 | 0.3 | 0.3 | 0.00 |
| 55-59 | 0.7 | 1.0 | -0.03 | 0.7 | 0.9 | -0.01 |
| 60-64 | 1.7 | 2.0 | -0.02 | 1.8 | 1.9 | -0.01 |
| 65-69 | 23.7 | 26.4 | -0.06 | 24.4 | 24.5 | 0.00 |
| 70-74 | 28.1 | 30.8 | -0.06 | 28.6 | 28.7 | 0.00 |
| 75-79 | 22.6 | 20.5 | 0.05 | 22.2 | 21.9 | 0.01 |
| 80-84 | 14.6 | 13.3 | 0.04 | 14.3 | 14.2 | 0.00 |
| 85-89 | 6.8 | 4.3 | 0.11 | 6.2 | 6.2 | 0.00 |
| 90-94 | 1.3 | 1.2 | 0.01 | 1.3 | 1.3 | 0.00 |
| 95-99 | 0.1 | 0.1 | 0.01 | 0.1 | 0.1 | 0.01 |
| 00-04 | 0.0 | <0.1 | -0.01 | 0.0 | <0.1 | -0.02 |
| Gender: female | 73.0 | 68.5 | 0.10 | 71.7 | 71.6 | 0.00 |
| Medical history: General |  |  |  |  |  |  |
| Acute respiratory disease | 27.5 | 26.5 | 0.02 | 27.3 | 27.3 | 0.00 |
| Attention deficit hyperactivity disorder | 0.2 | 0.2 | 0.00 | 0.2 | 0.2 | 0.00 |
| Chronic liver disease | 2.1 | 1.9 | 0.02 | 2.1 | 1.9 | 0.02 |
| Chronic obstructive lung disease | 15.7 | 16.2 | -0.01 | 15.7 | 16.2 | -0.01 |
| Crohn's disease | 0.5 | 1.3 | -0.08 | 0.6 | 1.1 | -0.05 |
| Dementia | 2.0 | 1.7 | 0.02 | 2.0 | 1.8 | 0.01 |
| Depressive disorder | 9.2 | 9.2 | 0.00 | 9.2 | 9.3 | 0.00 |
| Diabetes mellitus | 23.1 | 22.5 | 0.02 | 22.9 | 22.7 | 0.01 |
| Gastroesophageal reflux disease | 16.5 | 16.5 | 0.00 | 16.2 | 16.5 | -0.01 |
| Gastrointestinal hemorrhage | 4.8 | 5.1 | -0.01 | 4.8 | 4.9 | 0.00 |
| Human immunodeficiency virus infection | 0.1 | <0.1 | 0.01 | 0.1 | <0.1 | 0.01 |
| Hyperlipidemia | 41.3 | 39.3 | 0.04 | 40.7 | 41.3 | -0.01 |
| Hypertensive disorder | 61.8 | 59.4 | 0.05 | 61.1 | 61.5 | -0.01 |
| Lesion of liver | 1.0 | 1.0 | -0.01 | 1.0 | 1.0 | -0.01 |
| Lupus erythematosus | 0.9 | 0.2 | 0.09 | 0.8 | 0.2 | 0.09 |
| Obesity | 5.5 | 5.4 | 0.00 | 5.5 | 5.5 | 0.00 |
| Osteoarthritis | 62.0 | 59.6 | 0.05 | 61.2 | 61.9 | -0.01 |
| Pneumonia | 9.1 | 9.3 | 0.00 | 9.1 | 9.2 | 0.00 |
| Psoriasis | 2.3 | 4.9 | -0.14 | 2.7 | 3.3 | -0.04 |
| Renal impairment | 11.3 | 10.5 | 0.03 | 11.1 | 11.2 | 0.00 |
| Rheumatoid arthritis | 88.4 | 89.7 | -0.04 | 88.9 | 89.3 | -0.01 |
| Schizophrenia | 0.1 | <0.1 | 0.00 | 0.1 | 0.1 | -0.01 |
| Ulcerative colitis | 0.6 | 2.0 | -0.12 | 0.7 | 1.2 | -0.05 |
| Urinary tract infectious disease | 13.6 | 12.7 | 0.03 | 13.5 | 12.7 | 0.02 |
| Viral hepatitis C | 0.5 | 0.5 | -0.01 | 0.5 | 0.5 | 0.00 |
| Visual system disorder | 55.4 | 55.2 | 0.00 | 55.3 | 54.9 | 0.01 |
| Medical history: Cardiovascular disease |  |  |  |  |  |  |
| Atrial fibrillation | 10.6 | 9.5 | 0.04 | 10.4 | 10.2 | 0.00 |
| Cerebrovascular disease | 11.8 | 11.0 | 0.02 | 11.5 | 11.6 | 0.00 |
| Coronary arteriosclerosis | 19.7 | 19.8 | 0.00 | 19.6 | 19.3 | 0.01 |
| Heart disease | 44.3 | 44.4 | 0.00 | 44.0 | 44.7 | -0.01 |
| Heart failure | 10.1 | 9.9 | 0.01 | 10.0 | 10.4 | -0.01 |
| Ischemic heart disease | 10.1 | 10.1 | 0.00 | 10.1 | 10.3 | -0.01 |
| Peripheral vascular disease | 6.4 | 5.5 | 0.04 | 6.3 | 6.0 | 0.01 |
| Pulmonary embolism | 1.5 | 1.3 | 0.02 | 1.5 | 1.3 | 0.02 |
| Venous thrombosis | 3.4 | 3.4 | 0.00 | 3.3 | 3.5 | -0.01 |
| Medical history: Neoplasms |  |  |  |  |  |  |
| Hematologic neoplasm | 2.2 | 2.3 | 0.00 | 2.2 | 2.4 | -0.01 |
| Malignant lymphoma | 1.3 | 1.5 | -0.02 | 1.3 | 1.4 | -0.01 |
| Malignant neoplasm of anorectum | 0.4 | 0.4 | 0.00 | 0.4 | 0.4 | 0.01 |
| Malignant neoplastic disease | 18.5 | 19.8 | -0.03 | 18.7 | 18.9 | 0.00 |
| Malignant tumor of breast | 3.8 | 4.0 | -0.01 | 3.8 | 3.7 | 0.00 |
| Malignant tumor of colon | 0.8 | 0.7 | 0.02 | 0.9 | 0.6 | 0.03 |
| Malignant tumor of lung | 0.7 | 0.6 | 0.00 | 0.6 | 0.5 | 0.02 |
| Malignant tumor of urinary bladder | 1.0 | 1.2 | -0.02 | 1.0 | 1.0 | 0.00 |
| Primary malignant neoplasm of prostate | 2.4 | 2.8 | -0.03 | 2.5 | 2.4 | 0.00 |
| Medication use |  |  |  |  |  |  |
| Agents acting on the renin-angiotensin system | 48.1 | 47.1 | 0.02 | 47.9 | 48.2 | -0.01 |
| Antibacterials for systemic use | 46.3 | 49.0 | -0.05 | 47.0 | 46.2 | 0.02 |
| Antidepressants | 31.5 | 31.4 | 0.00 | 31.6 | 31.5 | 0.00 |
| Antiepileptics | 20.1 | 21.8 | -0.04 | 20.3 | 20.7 | -0.01 |
| Antiinflammatory and antirheumatic products | 44.0 | 44.6 | -0.01 | 44.1 | 44.9 | -0.02 |
| Antineoplastic agents | 32.0 | 40.6 | -0.18 | 34.8 | 33.0 | 0.04 |
| Antipsoriatics | 1.0 | 1.3 | -0.03 | 1.0 | 1.3 | -0.02 |
| Antithrombotic agents | 22.8 | 22.1 | 0.02 | 22.6 | 22.3 | 0.01 |
| Beta blocking agents | 39.4 | 39.4 | 0.00 | 39.3 | 39.1 | 0.00 |
| Calcium channel blockers | 30.1 | 28.5 | 0.04 | 29.6 | 30.4 | -0.02 |
| Diuretics | 47.1 | 45.3 | 0.04 | 46.7 | 46.3 | 0.01 |
| Drugs for acid related disorders | 44.6 | 45.6 | -0.02 | 44.6 | 45.1 | -0.01 |
| Drugs for obstructive airway diseases | 33.2 | 32.5 | 0.02 | 33.1 | 32.9 | 0.00 |
| Drugs used in diabetes | 17.4 | 16.9 | 0.02 | 17.4 | 16.9 | 0.01 |
| Immunosuppressants | 41.1 | 55.1 | -0.28 | 45.5 | 42.5 | 0.06 |
| Lipid modifying agents | 49.8 | 48.6 | 0.02 | 49.5 | 49.4 | 0.00 |
| Opioids | 43.6 | 44.0 | -0.01 | 43.8 | 43.2 | 0.01 |
| Psycholeptics | 34.6 | 34.1 | 0.01 | 34.5 | 33.6 | 0.02 |
| Psychostimulants, agents used for adhd and nootropics | 2.0 | 2.0 | 0.00 | 2.0 | 1.9 | 0.01 |

## OpenClaims

|  | **Before PS stratification** | | | **After PS stratification** | | |
| --- | --- | --- | --- | --- | --- | --- |
|  | **HCQ** | **SSZ** |  | **HCQ** | **SSZ** |  |
| **Characteristic** | **%** | **%** | **Std. diff** | **%** | **%** | **Std. diff** |
| Age group |  |  |  |  |  |  |
| 15-19 | 0.3 | 0.3 | -0.01 | 0.3 | 0.3 | 0.00 |
| 20-24 | 1.2 | 1.2 | -0.01 | 1.2 | 1.2 | 0.00 |
| 25-29 | 1.9 | 1.9 | 0.00 | 1.9 | 1.8 | 0.00 |
| 30-34 | 3.0 | 2.9 | 0.00 | 2.9 | 2.9 | 0.00 |
| 35-39 | 4.3 | 4.4 | -0.01 | 4.3 | 4.3 | 0.00 |
| 40-44 | 5.8 | 5.9 | 0.00 | 5.8 | 5.7 | 0.00 |
| 45-49 | 8.3 | 8.3 | 0.00 | 8.2 | 8.1 | 0.00 |
| 50-54 | 11.5 | 11.9 | -0.01 | 11.5 | 11.5 | 0.00 |
| 55-59 | 13.6 | 14.2 | -0.02 | 13.7 | 13.8 | 0.00 |
| 60-64 | 13.4 | 13.8 | -0.01 | 13.6 | 13.5 | 0.00 |
| 65-69 | 12.5 | 12.7 | 0.00 | 12.6 | 12.6 | 0.00 |
| 70-74 | 9.9 | 9.6 | 0.01 | 9.9 | 10.0 | 0.00 |
| 75-79 | 10.7 | 9.0 | 0.06 | 10.3 | 10.6 | -0.01 |
| 80-84 | 3.7 | 3.5 | 0.01 | 3.6 | 3.6 | 0.00 |
| 85-89 | 0.2 | 0.2 | 0.00 | 0.2 | 0.2 | 0.00 |
| Gender: female | 81.0 | 73.5 | 0.18 | 79.3 | 79.1 | 0.00 |
| Medical history: General |  |  |  |  |  |  |
| Acute respiratory disease | 20.1 | 19.2 | 0.02 | 19.9 | 19.9 | 0.00 |
| Attention deficit hyperactivity disorder | 0.7 | 0.7 | 0.00 | 0.7 | 0.7 | 0.00 |
| Chronic liver disease | 2.1 | 2.0 | 0.00 | 2.0 | 2.1 | 0.00 |
| Chronic obstructive lung disease | 7.6 | 7.7 | 0.00 | 7.6 | 7.8 | 0.00 |
| Crohn's disease | 0.4 | 1.6 | -0.11 | 0.5 | 0.9 | -0.04 |
| Dementia | 0.8 | 0.7 | 0.01 | 0.8 | 0.8 | 0.00 |
| Depressive disorder | 9.7 | 9.6 | 0.00 | 9.6 | 9.8 | -0.01 |
| Diabetes mellitus | 15.6 | 16.1 | -0.01 | 15.7 | 16.0 | -0.01 |
| Gastroesophageal reflux disease | 4.3 | 5.0 | -0.04 | 4.5 | 4.5 | 0.00 |
| Gastrointestinal hemorrhage | 2.4 | 2.5 | -0.01 | 2.4 | 2.4 | 0.00 |
| Human immunodeficiency virus infection | 0.2 | 0.2 | 0.00 | 0.2 | 0.2 | 0.00 |
| Hyperlipidemia | 26.0 | 25.8 | 0.01 | 26.0 | 26.4 | -0.01 |
| Hypertensive disorder | 35.0 | 34.5 | 0.01 | 34.9 | 35.2 | -0.01 |
| Lesion of liver | 0.8 | 0.7 | 0.01 | 0.8 | 0.8 | 0.00 |
| Lupus erythematosus | 1.1 | 0.3 | 0.10 | 0.9 | 0.5 | 0.05 |
| Obesity | 6.3 | 6.6 | -0.01 | 6.4 | 6.5 | 0.00 |
| Osteoarthritis | 36.0 | 37.0 | -0.02 | 36.1 | 36.9 | -0.02 |
| Pneumonia | 4.4 | 4.2 | 0.01 | 4.3 | 4.3 | 0.00 |
| Psoriasis | 1.9 | 5.4 | -0.19 | 2.2 | 3.3 | -0.06 |
| Renal impairment | 6.2 | 5.5 | 0.03 | 6.1 | 6.0 | 0.00 |
| Rheumatoid arthritis | 69.0 | 69.6 | -0.01 | 69.3 | 69.7 | -0.01 |
| Schizophrenia | 0.2 | 0.2 | 0.00 | 0.2 | 0.2 | 0.00 |
| Ulcerative colitis | 0.4 | 1.7 | -0.12 | 0.5 | 1.0 | -0.06 |
| Urinary tract infectious disease | 8.7 | 7.4 | 0.05 | 8.4 | 8.4 | 0.00 |
| Viral hepatitis C | 1.1 | 1.1 | 0.00 | 1.1 | 1.1 | 0.00 |
| Visual system disorder | 21.9 | 20.7 | 0.03 | 21.6 | 21.6 | 0.00 |
| Medical history: Cardiovascular disease |  |  |  |  |  |  |
| Atrial fibrillation | 3.6 | 3.4 | 0.01 | 3.6 | 3.6 | 0.00 |
| Cerebrovascular disease | 3.4 | 2.8 | 0.03 | 3.2 | 3.3 | 0.00 |
| Coronary arteriosclerosis | 6.8 | 6.8 | 0.00 | 6.8 | 6.9 | 0.00 |
| Heart disease | 18.3 | 17.3 | 0.03 | 18.1 | 18.3 | 0.00 |
| Heart failure | 4.0 | 3.7 | 0.02 | 3.9 | 4.0 | 0.00 |
| Ischemic heart disease | 3.5 | 3.4 | 0.01 | 3.5 | 3.6 | 0.00 |
| Peripheral vascular disease | 9.7 | 9.1 | 0.02 | 9.6 | 9.6 | 0.00 |
| Pulmonary embolism | 0.8 | 0.7 | 0.01 | 0.8 | 0.8 | 0.00 |
| Venous thrombosis | 1.5 | 1.4 | 0.01 | 1.5 | 1.5 | 0.00 |
| Medical history: Neoplasms |  |  |  |  |  |  |
| Hematologic neoplasm | 0.9 | 0.9 | 0.00 | 0.9 | 1.0 | 0.00 |
| Malignant lymphoma | 0.6 | 0.6 | 0.00 | 0.6 | 0.6 | 0.00 |
| Malignant neoplasm of anorectum | 0.1 | 0.1 | 0.00 | 0.1 | 0.1 | 0.00 |
| Malignant neoplastic disease | 7.2 | 7.2 | 0.00 | 7.2 | 7.4 | 0.00 |
| Malignant tumor of breast | 1.8 | 1.6 | 0.01 | 1.7 | 1.7 | 0.00 |
| Malignant tumor of colon | 0.3 | 0.3 | 0.00 | 0.3 | 0.3 | 0.00 |
| Malignant tumor of lung | 0.3 | 0.3 | 0.00 | 0.3 | 0.3 | 0.00 |
| Malignant tumor of urinary bladder | 0.2 | 0.3 | -0.01 | 0.3 | 0.3 | 0.00 |
| Primary malignant neoplasm of prostate | 0.6 | 0.8 | -0.02 | 0.7 | 0.7 | 0.00 |
| Medication use |  |  |  |  |  |  |
| Agents acting on the renin-angiotensin system | 30.8 | 33.0 | -0.05 | 31.4 | 31.4 | 0.00 |
| Antibacterials for systemic use | 36.1 | 38.8 | -0.06 | 36.8 | 36.6 | 0.00 |
| Antidepressants | 34.5 | 35.7 | -0.03 | 34.7 | 34.8 | 0.00 |
| Antiepileptics | 24.0 | 26.4 | -0.05 | 24.5 | 24.6 | 0.00 |
| Antiinflammatory and antirheumatic products | 41.0 | 45.2 | -0.08 | 42.0 | 41.9 | 0.00 |
| Antineoplastic agents | 31.6 | 38.0 | -0.14 | 33.3 | 33.4 | 0.00 |
| Antipsoriatics | 0.7 | 1.0 | -0.03 | 0.8 | 0.9 | -0.01 |
| Antithrombotic agents | 12.9 | 12.8 | 0.00 | 12.9 | 12.9 | 0.00 |
| Beta blocking agents | 23.1 | 24.1 | -0.02 | 23.3 | 23.5 | 0.00 |
| Calcium channel blockers | 17.1 | 17.1 | 0.00 | 17.1 | 17.1 | 0.00 |
| Diuretics | 28.1 | 28.8 | -0.02 | 28.3 | 28.3 | 0.00 |
| Drugs for acid related disorders | 35.3 | 37.5 | -0.04 | 35.8 | 35.8 | 0.00 |
| Drugs for obstructive airway diseases | 29.0 | 31.0 | -0.04 | 29.5 | 29.5 | 0.00 |
| Drugs used in diabetes | 14.0 | 15.4 | -0.04 | 14.3 | 14.6 | -0.01 |
| Immunosuppressants | 41.9 | 53.4 | -0.23 | 44.7 | 44.7 | 0.00 |
| Lipid modifying agents | 29.0 | 31.5 | -0.05 | 29.6 | 29.8 | -0.01 |
| Opioids | 34.8 | 37.8 | -0.06 | 35.4 | 35.5 | 0.00 |
| Psycholeptics | 29.5 | 30.8 | -0.03 | 29.7 | 29.9 | 0.00 |
| Psychostimulants, agents used for adhd and nootropics | 4.5 | 4.8 | -0.01 | 4.5 | 4.6 | 0.00 |

## OptumEHR

|  | **Before PS stratification** | | | **After PS stratification** | | |
| --- | --- | --- | --- | --- | --- | --- |
|  | **HCQ** | **SSZ** |  | **HCQ** | **SSZ** |  |
| **Characteristic** | **%** | **%** | **Std. diff** | **%** | **%** | **Std. diff** |
| Age group |  |  |  |  |  |  |
| 15-19 | 0.2 | 0.2 | -0.01 | 0.2 | 0.2 | -0.01 |
| 20-24 | 1.0 | 1.0 | 0.00 | 1.0 | 1.0 | 0.00 |
| 25-29 | 1.8 | 1.9 | -0.01 | 1.8 | 1.9 | -0.01 |
| 30-34 | 3.1 | 3.1 | 0.00 | 3.1 | 3.0 | 0.00 |
| 35-39 | 4.2 | 4.5 | -0.02 | 4.2 | 4.2 | 0.00 |
| 40-44 | 5.9 | 5.7 | 0.01 | 5.9 | 5.5 | 0.02 |
| 45-49 | 7.9 | 8.3 | -0.01 | 7.9 | 7.9 | 0.00 |
| 50-54 | 11.5 | 11.7 | -0.01 | 11.6 | 11.4 | 0.00 |
| 55-59 | 13.8 | 14.8 | -0.03 | 14.0 | 14.2 | -0.01 |
| 60-64 | 13.3 | 13.9 | -0.02 | 13.5 | 13.5 | 0.00 |
| 65-69 | 12.1 | 12.6 | -0.02 | 12.2 | 12.6 | -0.01 |
| 70-74 | 10.0 | 9.7 | 0.01 | 10.0 | 9.9 | 0.00 |
| 75-79 | 8.1 | 7.0 | 0.04 | 7.9 | 7.9 | 0.00 |
| 80-84 | 5.4 | 4.3 | 0.06 | 5.2 | 5.3 | 0.00 |
| 85-89 | 1.5 | 1.2 | 0.03 | 1.5 | 1.4 | 0.01 |
| Gender: female | 79.9 | 72.4 | 0.18 | 78.2 | 78.5 | -0.01 |
| Race |  |  |  |  |  |  |
| race = Asian | 1.5 | 1.1 | 0.04 | 1.5 | 1.4 | 0.01 |
| race = Black or African American | 10.0 | 8.6 | 0.05 | 9.8 | 9.5 | 0.01 |
| race = White | 81.4 | 83.3 | -0.05 | 81.7 | 82.0 | -0.01 |
| Ethnicity |  |  |  |  |  |  |
| ethnicity = Hispanic or Latino | 5.3 | 6.0 | -0.03 | 5.4 | 5.4 | 0.00 |
| Medical history: General |  |  |  |  |  |  |
| Acute respiratory disease | 17.8 | 18.6 | -0.02 | 17.9 | 17.8 | 0.00 |
| Attention deficit hyperactivity disorder | 0.9 | 0.8 | 0.01 | 0.9 | 0.8 | 0.01 |
| Chronic liver disease | 2.7 | 3.0 | -0.02 | 2.8 | 2.9 | 0.00 |
| Chronic obstructive lung disease | 8.5 | 9.2 | -0.03 | 8.7 | 8.9 | -0.01 |
| Crohn's disease | 0.5 | 1.7 | -0.12 | 0.6 | 1.0 | -0.04 |
| Dementia | 1.1 | 1.0 | 0.01 | 1.0 | 1.1 | -0.01 |
| Depressive disorder | 15.7 | 15.5 | 0.00 | 15.6 | 15.4 | 0.00 |
| Diabetes mellitus | 14.9 | 15.1 | -0.01 | 15.0 | 15.2 | 0.00 |
| Gastroesophageal reflux disease | 18.3 | 18.3 | 0.00 | 18.3 | 18.0 | 0.01 |
| Gastrointestinal hemorrhage | 2.1 | 2.5 | -0.02 | 2.1 | 2.2 | 0.00 |
| Human immunodeficiency virus infection | 0.1 | 0.2 | -0.01 | 0.1 | 0.2 | -0.01 |
| Hyperlipidemia | 30.3 | 31.5 | -0.03 | 30.5 | 30.4 | 0.00 |
| Hypertensive disorder | 38.8 | 39.0 | 0.00 | 38.8 | 39.1 | -0.01 |
| Lesion of liver | 1.1 | 0.9 | 0.02 | 1.0 | 1.0 | 0.01 |
| Lupus erythematosus | 1.0 | 0.3 | 0.09 | 0.9 | 0.5 | 0.04 |
| Obesity | 11.3 | 12.3 | -0.03 | 11.4 | 11.7 | -0.01 |
| Osteoarthritis | 35.0 | 36.8 | -0.04 | 35.3 | 35.4 | 0.00 |
| Pneumonia | 4.9 | 5.1 | -0.01 | 5.0 | 4.9 | 0.00 |
| Psoriasis | 1.7 | 4.6 | -0.17 | 2.0 | 2.7 | -0.04 |
| Renal impairment | 7.6 | 6.9 | 0.03 | 7.4 | 7.5 | 0.00 |
| Rheumatoid arthritis | 84.8 | 84.8 | 0.00 | 84.9 | 85.1 | -0.01 |
| Schizophrenia | 0.2 | 0.1 | 0.01 | 0.2 | 0.2 | 0.00 |
| Ulcerative colitis | 0.4 | 1.5 | -0.12 | 0.5 | 0.8 | -0.04 |
| Urinary tract infectious disease | 7.5 | 6.4 | 0.04 | 7.3 | 7.0 | 0.01 |
| Viral hepatitis C | 1.2 | 1.5 | -0.02 | 1.3 | 1.4 | -0.01 |
| Visual system disorder | 13.7 | 13.9 | 0.00 | 13.7 | 13.3 | 0.01 |
| Medical history: Cardiovascular disease |  |  |  |  |  |  |
| Atrial fibrillation | 4.5 | 4.0 | 0.03 | 4.5 | 4.5 | 0.00 |
| Cerebrovascular disease | 3.1 | 3.1 | 0.00 | 3.1 | 3.0 | 0.00 |
| Coronary arteriosclerosis | 8.3 | 8.5 | -0.01 | 8.3 | 8.4 | 0.00 |
| Heart disease | 20.8 | 20.4 | 0.01 | 20.7 | 21.1 | -0.01 |
| Heart failure | 4.7 | 4.4 | 0.01 | 4.7 | 4.8 | -0.01 |
| Ischemic heart disease | 4.4 | 4.5 | -0.01 | 4.4 | 4.4 | 0.00 |
| Peripheral vascular disease | 2.6 | 2.4 | 0.01 | 2.6 | 2.4 | 0.01 |
| Pulmonary embolism | 1.0 | 1.1 | -0.01 | 1.0 | 1.0 | -0.01 |
| Venous thrombosis | 1.9 | 2.0 | -0.01 | 1.9 | 2.0 | -0.01 |
| Medical history: Neoplasms |  |  |  |  |  |  |
| Hematologic neoplasm | 1.1 | 1.2 | -0.01 | 1.1 | 1.2 | 0.00 |
| Malignant lymphoma | 0.6 | 0.7 | -0.01 | 0.6 | 0.7 | -0.01 |
| Malignant neoplasm of anorectum | 0.2 | 0.2 | 0.01 | 0.2 | 0.2 | 0.01 |
| Malignant neoplastic disease | 8.5 | 8.8 | -0.01 | 8.5 | 8.7 | -0.01 |
| Malignant tumor of breast | 1.9 | 1.7 | 0.02 | 1.9 | 1.9 | 0.00 |
| Malignant tumor of colon | 0.4 | 0.4 | 0.00 | 0.4 | 0.4 | 0.00 |
| Malignant tumor of lung | 0.4 | 0.4 | -0.01 | 0.4 | 0.4 | -0.01 |
| Malignant tumor of urinary bladder | 0.2 | 0.4 | -0.03 | 0.2 | 0.3 | -0.02 |
| Primary malignant neoplasm of prostate | 0.6 | 0.9 | -0.03 | 0.7 | 0.7 | 0.00 |
| Medication use |  |  |  |  |  |  |
| Agents acting on the renin-angiotensin system | 30.5 | 31.8 | -0.03 | 30.8 | 31.3 | -0.01 |
| Antibacterials for systemic use | 28.0 | 30.2 | -0.05 | 28.3 | 28.1 | 0.00 |
| Antidepressants | 35.9 | 35.9 | 0.00 | 35.9 | 36.1 | -0.01 |
| Antiepileptics | 23.9 | 25.0 | -0.03 | 24.2 | 24.4 | -0.01 |
| Antiinflammatory and antirheumatic products | 43.8 | 47.3 | -0.07 | 44.5 | 44.3 | 0.00 |
| Antineoplastic agents | 34.7 | 37.3 | -0.05 | 35.4 | 36.6 | -0.03 |
| Antipsoriatics | 0.6 | 0.7 | -0.01 | 0.6 | 0.6 | 0.00 |
| Antithrombotic agents | 32.9 | 31.7 | 0.03 | 32.7 | 33.1 | -0.01 |
| Beta blocking agents | 26.7 | 27.1 | -0.01 | 26.8 | 27.3 | -0.01 |
| Calcium channel blockers | 17.1 | 16.8 | 0.01 | 17.1 | 17.4 | -0.01 |
| Diuretics | 29.2 | 28.5 | 0.02 | 29.1 | 29.2 | 0.00 |
| Drugs for acid related disorders | 45.1 | 45.7 | -0.01 | 45.2 | 45.4 | 0.00 |
| Drugs for obstructive airway diseases | 34.5 | 35.5 | -0.02 | 34.7 | 34.8 | 0.00 |
| Drugs used in diabetes | 15.1 | 15.9 | -0.02 | 15.3 | 15.8 | -0.02 |
| Immunosuppressants | 47.4 | 55.7 | -0.17 | 49.2 | 50.3 | -0.02 |
| Lipid modifying agents | 30.2 | 31.9 | -0.04 | 30.6 | 30.9 | -0.01 |
| Opioids | 39.3 | 41.5 | -0.04 | 39.7 | 40.1 | -0.01 |
| Psycholeptics | 35.1 | 35.4 | -0.01 | 35.1 | 35.2 | 0.00 |
| Psychostimulants, agents used for adhd and nootropics | 4.3 | 4.1 | 0.01 | 4.2 | 4.0 | 0.01 |

# Study diagnostics

Preference score overlap, covariate balance, and empirical calibration plots are reported below. The preference score is a transformation of the propensity score that adjusts for differences in the sizes of the two exposure cohorts in a pairwise comparison. A higher overlap indicates patients in the two cohorts were more similar in terms of their predicted probability of receiving one exposure relative to the other. In the covariate balance plots, each dot represents the standardizes difference of means for a single covariate before and after propensity score stratification. The empirical calibration plots show effect estimates for the negative controls where the true hazard ratio is expected to equal 1. Estimates below the diagonal dashed lines are statistically significantly different (alpha = 0.05) from the true effect size. A well-calibrated estimator should produce the true effect sizes within the 95 percent confidence interval 95 percent of the time. Negative control estimates are not reported if fewer than 5 events are observed. *On-treatment* time-at-risk was unavailable in the OptumEHR databases so no negative control effects were estimated.

## Diagnostics (AmbEMR, CCAE, Clinformatics, CPRD, DAGermany)


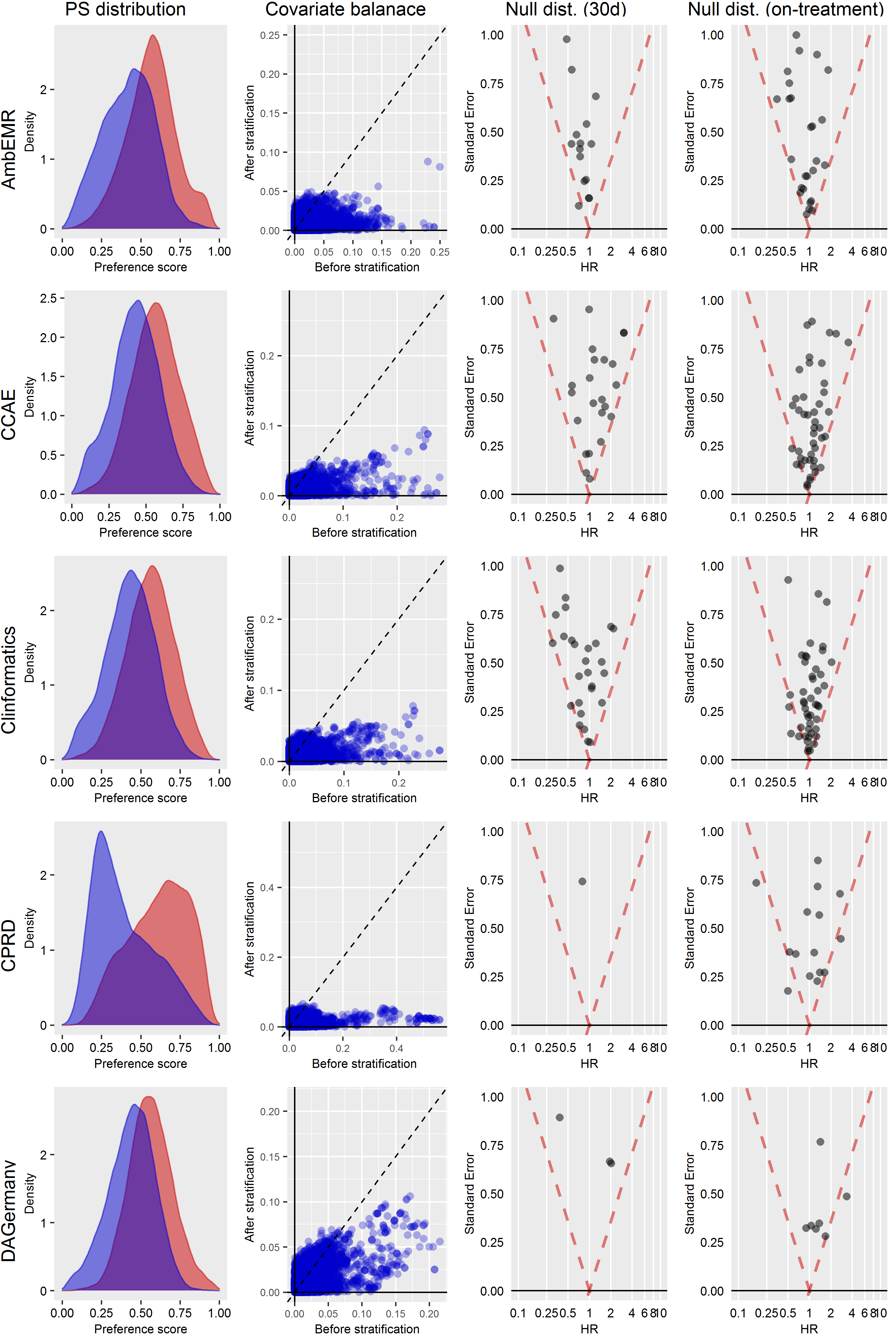


## Diagnostics (IMRD, MDCD, MDCR, OpenClaims, OptumEHR)


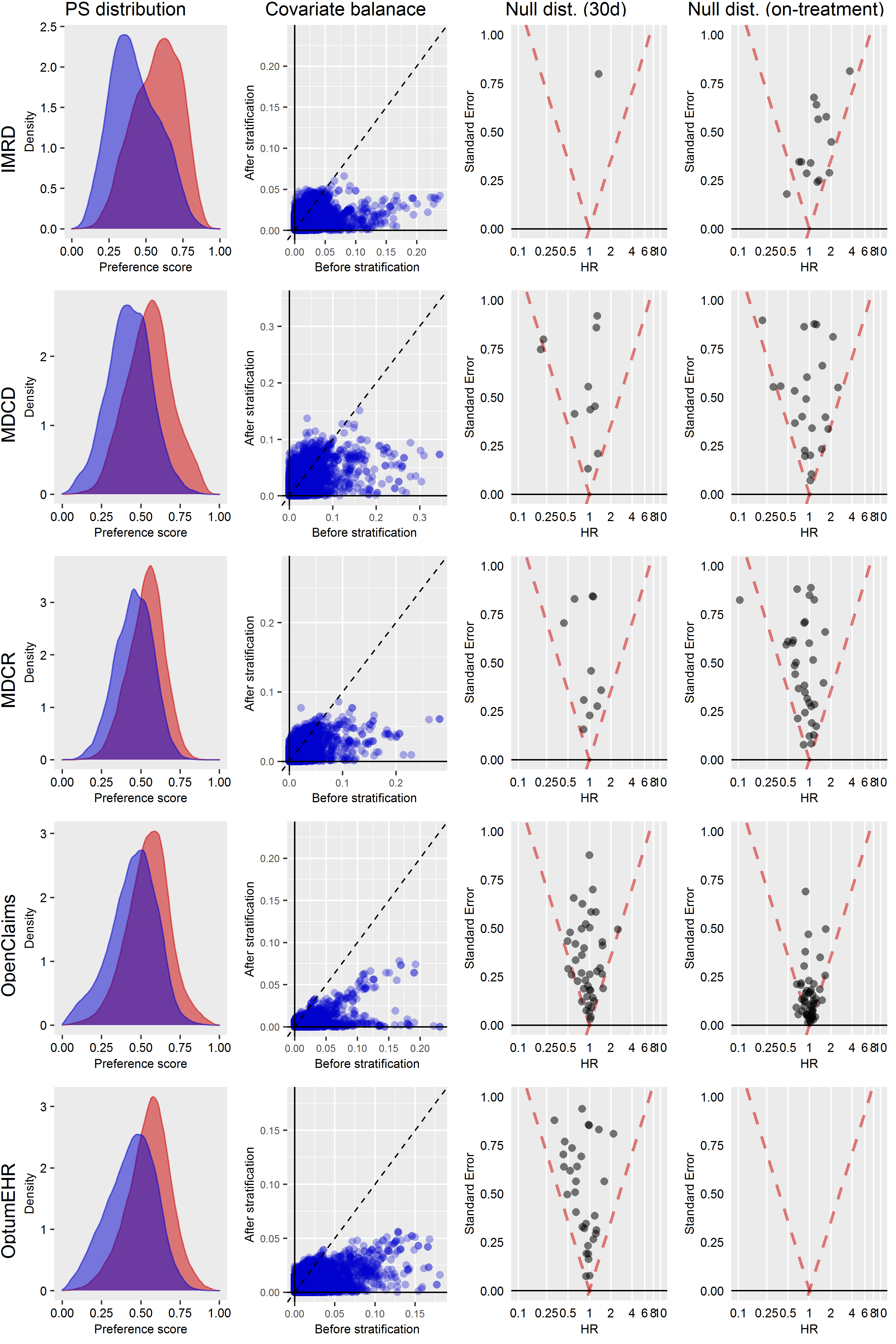


# Incidence rates

Patient counts, event counts, and incidence rates per 1,000 person-years are reported below for HCQ vs SSZ during the two follow-up periods. Note that the incidence rate does not account for stratification.

## 30-day follow-up

|  |  | Patients |  | TAR |  | Events |  | IR |  |  |
| --- | --- | --- | --- | --- | --- | --- | --- | --- | --- | --- |
| Outcome | Database | T | C | T | C | T | C | T | C | MDRR |
| Depression | AmbEMR | 55,793 | 15,092 | 4,571 | 1,237 | 155 | 29 | 33.91 | 23.44 | 1.66 |
|  | CCAE | 66,440 | 22,449 | 5,395 | 1,822 | 79 | 28 | 14.64 | 15.36 | 1.87 |
|  | Clinformatics | 51,676 | 16,812 | 4,190 | 1,362 | 84 | 41 | 20.05 | 30.09 | 1.79 |
|  | CPRD | 9,160 | 11,348 | 749 | 930 | <5 | 8 | <6.67 | 8.60 | >4.77 |
|  | DAGermany | 3,937 | 5,109 | 322 | 419 | <5 | 12 | <15.48 | 28.63 | >3.94 |
|  | IMRD | 8,844 | 8,456 | 723 | 692 | <5 | 6 | <6.91 | 8.67 | >5.42 |
|  | MDCD | 7,950 | 2,286 | 647 | 185 | 14 | 6 | 21.61 | 32.29 | 4.50 |
|  | MDCR | 15,735 | 5,275 | 1,282 | 429 | 13 | 6 | 10.14 | 13.98 | 4.40 |
|  | OpenClaims | 620,081 | 183,312 | 50,893 | 15,046 | 654 | 161 | 12.85 | 10.70 | 1.26 |
|  | OptumEHR | 78,528 | 20,244 | 6,348 | 1,637 | 321 | 66 | 50.56 | 40.30 | 1.42 |
|  | Meta-analysis | 918,144 | 290,383 | 75,126 | 23,764 | <1,335 | 363 | <17.77 | 15.28 | >1.17 |
| Suicide and suicidal ideation | CCAE | 66,533 | 22,471 | 5,405 | 1,825 | 12 | <5 | 2.22 | <2.74 | >11.44 |
|  | Clinformatics | 51,807 | 16,843 | 4,203 | 1,366 | 12 | <5 | 2.85 | <3.66 | >11.72 |
|  | CPRD | 9,167 | 11,358 | 750 | 931 | <5 | <5 | <6.66 | <5.37 | >Inf |
|  | IMRD | 8,852 | 8,460 | 723 | 692 | <5 | <5 | <6.91 | <7.22 | >Inf |
|  | MDCD | 7,980 | 2,296 | 650 | 186 | <5 | <5 | <7.68 | <26.78 | >Inf |
|  | OpenClaims | 621,067 | 183,550 | 50,999 | 15,072 | 34 | 8 | 0.67 | 0.53 | 2.80 |
|  | OptumEHR | 79,903 | 20,480 | 6,474 | 1,659 | 18 | 8 | 2.78 | 4.82 | 3.91 |
|  | Meta-analysis | 845,309 | 265,458 | 69,208 | 21,734 | <91 | <41 | <1.31 | <1.89 | >2.53 |
| Hospitalization for psychosis | OpenClaims | 620,964 | 183,527 | 50,988 | 15,069 | 95 | 27 | 1.86 | 1.79 | 1.83 |
|  | OptumEHR | 79,994 | 20,508 | 6,482 | 1,661 | <5 | <5 | <0.77 | <3.01 | >Inf |
|  | Meta-analysis | 700,958 | 204,035 | 57,470 | 16,731 | <100 | <32 | <1.74 | <1.91 | >2.25 |

## On-treatment follow-up

|  |  | Patients |  | TAR |  | Events |  | IR |  |  |
| --- | --- | --- | --- | --- | --- | --- | --- | --- | --- | --- |
| Outcome | Database | T | C | T | C | T | C | T | C | MDRR |
| Depression | AmbEMR | 55,793 | 15,092 | 18,043 | 5,579 | 320 | 80 | 17.74 | 14.34 | 1.41 |
|  | CCAE | 66,440 | 22,449 | 65,249 | 14,578 | 557 | 137 | 8.54 | 9.40 | 1.28 |
|  | Clinformatics | 51,676 | 16,812 | 52,863 | 11,866 | 657 | 178 | 12.43 | 15.00 | 1.25 |
|  | CPRD | 9,160 | 11,348 | 18,063 | 26,115 | 36 | 94 | 1.99 | 3.60 | 1.64 |
|  | DAGermany | 3,937 | 5,109 | 2,585 | 3,561 | 40 | 70 | 15.47 | 19.66 | 1.71 |
|  | IMRD | 8,844 | 8,456 | 17,274 | 18,765 | 38 | 51 | 2.20 | 2.72 | 1.81 |
|  | MDCD | 7,950 | 2,286 | 5,691 | 1,284 | 90 | 13 | 15.81 | 10.12 | 1.94 |
|  | MDCR | 15,735 | 5,275 | 18,059 | 4,098 | 97 | 38 | 5.37 | 9.27 | 1.74 |
|  | OpenClaims | 620,081 | 183,312 | 859,978 | 171,406 | 4,810 | 957 | 5.59 | 5.58 | 1.09 |
|  | Meta-analysis | 839,616 | 270,139 | 1,057,807 | 257,256 | 6,645 | 1,618 | 6.28 | 6.29 | 1.07 |
| Suicide and suicidal ideation | AmbEMR | 57,660 | 15,357 | 18,837 | 5,704 | 6 | <5 | 0.32 | <0.88 | >967.23 |
|  | CCAE | 66,533 | 22,471 | 65,931 | 14,687 | 81 | 28 | 1.23 | 1.91 | 1.85 |
|  | Clinformatics | 51,807 | 16,843 | 53,786 | 11,999 | 97 | 30 | 1.80 | 2.50 | 1.78 |
|  | CPRD | 9,167 | 11,358 | 18,127 | 26,379 | 7 | 9 | 0.39 | 0.34 | 4.09 |
|  | IMRD | 8,852 | 8,460 | 17,362 | 18,928 | 8 | 6 | 0.46 | 0.32 | 4.47 |
|  | MDCD | 7,980 | 2,296 | 5,764 | 1,278 | 56 | 18 | 9.71 | 14.08 | 2.19 |
|  | MDCR | 15,752 | 5,278 | 18,168 | 4,125 | 15 | 6 | 0.83 | 1.45 | 4.10 |
|  | OpenClaims | 621,067 | 183,550 | 869,744 | 172,782 | 321 | 89 | 0.37 | 0.52 | 1.39 |
|  | Meta-analysis | 838,818 | 265,613 | 1,067,722 | 255,886 | 591 | <191 | 0.55 | <0.75 | >1.39 |
| Hospitalization for psychosis | OpenClaims | 620,964 | 183,527 | 868,568 | 172,595 | 1,108 | 221 | 1.28 | 1.28 | 1.20 |
